# Model‐Guided Rational Construction of *Escherichia coli* Synthetic Consortia for Enhanced 2‐Methylbutyric Acid Production

**DOI:** 10.1002/advs.202416272

**Published:** 2025-05-05

**Authors:** Yu Liu, Boyuan Xue, Shaojie Wang, Haijia Su

**Affiliations:** ^1^ State Key Laboratory of Green Biomanufacturing National Energy R&D Center for Biorefinery Beijing Key Laboratory of Green Chemicals Biomanufacturing Beijing Synthetic Bio‐manufacturing Technology Innovation Center Beijing 102209 P. R. China

**Keywords:** co‐culture, metabolic engineering, metabolic network model, rational construction, synthetic consortia

## Abstract

Synthetic consortia represent an innovative and effective platform that can significantly alleviate the metabolic burden on host organisms and enable flexible regulation of biosynthetic pathways. However, designing a stable synthetic consortium remains a significant challenge. In this study, a novel citramalate ‐derived pathway is first developed for 2‐methylbutyric acid (2MBA) biosynthesis in an *E. coli* mono‐culture system, achieving a titer of 678.78 ± 49.04 mg L^−1^. Furthermore, it employs a CulECpy model‐guided strategy to design and optimize the division of labor within *E. coli* synthetic consortia, predicting the optimal pathway allocation for improved 2MBA production. The best‐performing consortium, using 2‐keto‐3‐methylvalerate (KMV) as a single node, achieved 1817.03 ± 103.73 mg L^−1^ of 2MBA, a 28‐fold increase over the initial mono‐culture strain, with the highest reported yield of 0.091 g/g glucose. This work demonstrates the effectiveness of synthetic consortia and model‐guided pathway optimization for improving high‐value products, a versatile strategy that can be applied to the production of other valuable metabolites.

## Graphical abstract

We developed a citramalate‐derived pathway for 2‐methylbutyric acid production in *E. coli* mono‐culture and consortia. The optimized consortium, using 2‐keto‐3‐methylvalerate as a node, achieved 1817.03 ± 103.73 mg L^−1^ 2‐methylbutyric acid, a 28‐fold increase from the initial strain, with a yield of 0.091 g/g glucose. This model‐driven framework advances synthetic consortia for high‐value chemical production.

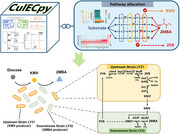

## 1 Introduction

Microorganisms harbor a wide variety of catalytic enzymes that create favorable conditions and environments for diverse biofuel synthesis.^[^ ^1^, ^2^ ^]^ Rapid advances in synthetic biology and metabolic engineering have significantly enhanced the ability to modify microorganisms, enabling them to synthesize advanced biofuels with tunable physical and combustion properties.^[^ ^3^, ^4^ ^]^ Short branched‐chain fatty acids (SBCFAs), such as isobutyric acid, 3‐methylbutyric acid, and 2‐methylbutyric acid (2MBA), are carboxylic acids containing 4 to 6 carbon atoms with methyl branches. Compared with straight‐chain fatty acids, branched‐chain fuels possess unique physicochemical properties, such as lower melting point, lower freezing point, and better oxidation stability, making them ideal for practical biofuel applications.^[^ ^5^, ^6^ ^]^ Furthermore, SBCFAs are crucial precursors in the manufacturing, pharmaceutical, and food industries, and are considered as multifunctional platform chemicals with high market demand.^[^ ^7^, ^8^, ^9^ ^]^

Currently, *de novo* synthesis of 2MBA from glucose are primarily derived from threonine pathway combined with Ehrlich pathway.^[^ ^10^, ^11^ ^]^ Initially, threonine is synthesized through the threonine pathway, and is then converted into 2‐ketobutyrate (2KB) via threonine deaminase. Subsequently, 2KB is processed through the branched‐chain amino acid pathway, leading to the formation of 2‐keto‐3‐methylvalerate (KMV). Finally, 2MBA is produced from KMV by employing the Ehrlich pathway with a 2‐keto acid decarboxylase (KDC) and aldehyde dehydrogenase (ALD). For example, Yu et al. illustrated that *Saccharomyces cerevisiae* produced 387.40 mg L^−1^ SBCFAs from yeast extract, peptone and dextrose via combinatorial metabolic engineering approach.^[^ ^10^ ^]^ However, the endogenous threonine synthesis is relatively complex and susceptible to negative feedback regulation. Moreover, the 2KB generated by threonine pathway requires significant consumption of ATP and NADPH, thereby imposing a substantial metabolic burden on the host organism.

Compared to the threonine pathway, the citramalate pathway is a promising choice due to its ability to complete 2‐ketobutyrate (2KB) synthesis in only three enzymatic steps, which has been extensively used for biosynthesizing propanol, butanol, and poly(3‐hydroxybutyrate‐co‐3‐hydroxyvalerate).^[^ ^12^, ^13^, ^14^, ^15^ ^]^ In the citramalate pathway, citramalate is synthesized by condensing pyruvate (PYR) with acetyl‐CoA, and then converted into 2KB by two enzymatic steps catalyzed by LeuBCD.^[^ ^15^, ^16^, ^17^, ^18^ ^]^ This pathway only avoids negative feedback regulation caused by amino acid synthesis but also simplifies 2KB synthetic pathway. Although simplifying the biosynthetic pathways can reduce cellular metabolic burden to some extent and enhance biosynthetic efficiency, achieving perfect metabolic balance within a single strain requires comprehensive coordination of various metabolic pathways and regulatory mechanisms.

Synthetic consortium, composed of two or more strains with division of labor, has emerged as an efficient platform to address metabolic burden in biosynthesis and balance metabolic flux.^[^ ^19^, ^20^, ^21^, ^22^ ^]^ By co‐culturing strains with distinct functions, synthetic consortium can distribute complex biosynthetic tasks across different functional strains to produce the desired product.^[^ ^23^, ^24^, ^25^ ^]^ In a synthetic consortium, metabolic pathways and functions are rationally designed and assigned to suitable microbial hosts, effectively converting complex substrates and intermediates into final products.^[^ ^21^, ^26^, ^27^ ^]^ However, engineering and optimizing synthetic consortia in such complex systems can be challenging, requiring tedious trial‐and‐error experiments to achieve the ideal balance of metabolic pathways within the consortium.

Model‐based methodologies offer a cost‐effective approach optimize biosynthetic systems by performing bulk simulations, forecasting experimental outcomes, and providing strategic insights for system optimization. Genome‐scale metabolic models (GEMs) have used in metabolic engineering for mono‐culture systems. Although metabolic models like OptCom, BacArena, and FLYCOP^[^ ^28^, ^29^, ^30^ ^]^ have been developed for synthetic consortia, they mainly address complex interspecies interactions. These methods fail to provide systematic guidance for the rational construction of synthetic consortia, and rarely offer de novo guidance for the experimental construction of a co‐cultivation synthesis system. Recently, we develeloped a co‐cultivated Enzyme Constraint metabolic network model (CulECpy)^[^ ^31^ ^]^ based on the concept of “independent + global”, which enables the prediction of optimal pathway allocation strategies for complex biosynthetic processes.

In this study, we developed a synthetic consortium approach for enhanced 2MBA biosynthesis by combining metabolic engineering with a model‐guided design strategy. Initially, an *E. coli* mono‐culture using a citramalate‐derived pathway was engineered, increasing 2MBA titer gradually to 678.78 ± 49.04 mg L^−1^. To further optimize production, we employed the CulECpy model to predict and design the optimal distribution of metabolic tasks between two strains. The resulting *E. coli* consortium, with KMV as the key intermediate, achieved a substantial increase in 2MBA production, reaching 1817.03 ± 103.73 mg L^−1^, with the highest yield of 0.091 g/g of glucose. This approach not only highlights the potential of synthetic consortia for improving biosynthetic processes but also demonstrates how model‐guided strategies can streamline the design and optimization of complex metabolic networks for high‐value product synthesis.

## 2 Results

### 2.1 Design and Construction of 2MBA Biosynthetic Pathway in Mono‐Culture

**Figure 1:**
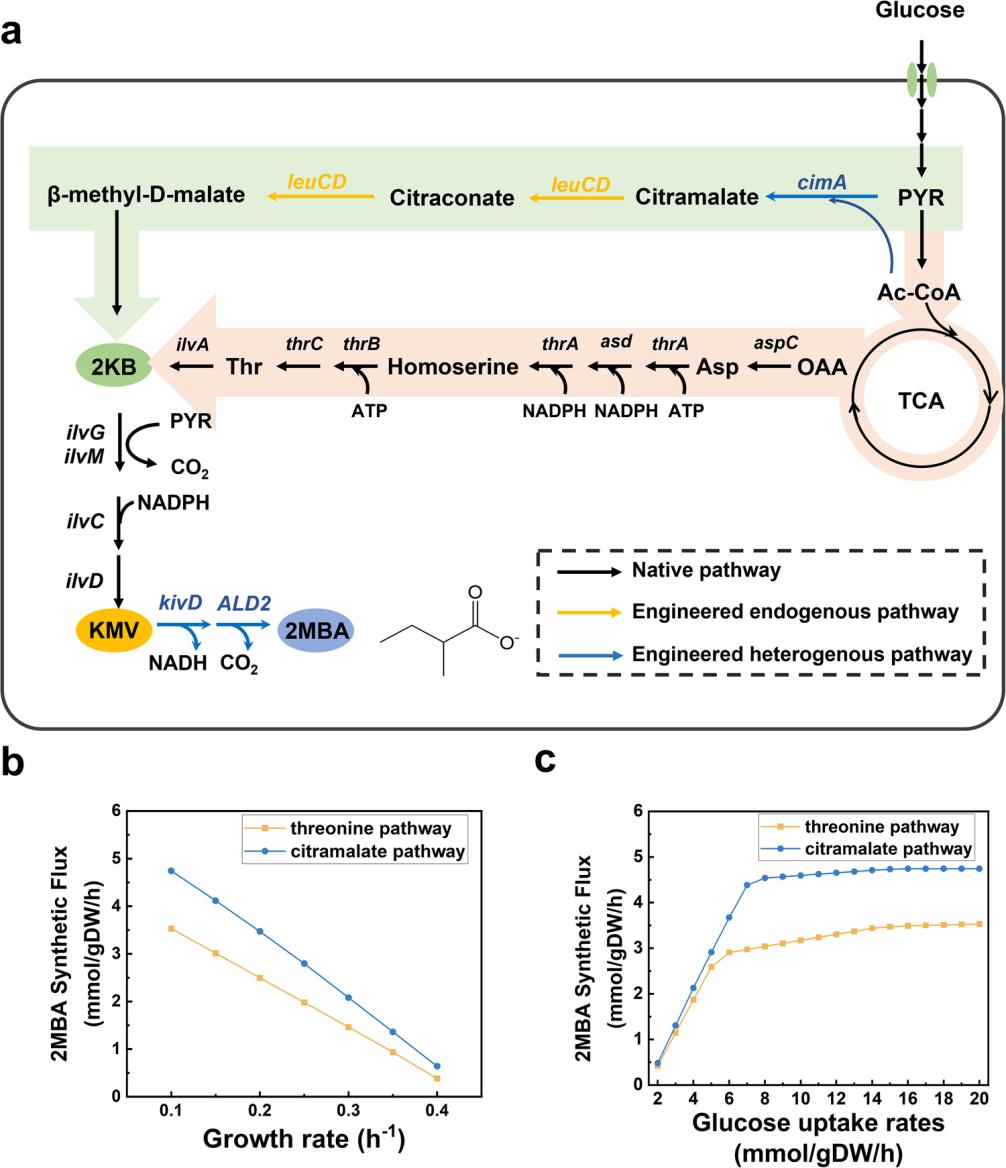
Schemes of citramalate‐derived pathway and threonine‐derived pathway for de novo production of 2‐methylbutyric acid (2MBA) from glucose. a) Schematic overview of 2MBA biosynthesis. The pathways highlighted with a green background represent the citramalate‐derived pathway, while the pathways with an orange background represent the threonine‐derived pathway. PYR, pyruvate; 2KB, 2‐ketobutyrate; KMV, 2‐keto‐3‐methyl‐valerate; Ac‐CoA, acetyl‐CoA; OAA, oxalacetic acid; Asp, aspartic acid; Thr, threonine; 2MBA, 2‐methylbutyric acid. Genome‐scale metabolic network models (GEMs) were further used to simulate and compare the two pathways b) at different growth rates and c) glucose uptake rates by enzyme‐constrained models.

In this study, two distinct synthetic pathways for the biosynthesis of 2‐methylbutyric acid (2MBA) were explored, i.e., the endogenous threonine pathway and the heterogenous citramalate pathway (**Figure** **1**a). The citramalate pathway demonstrated several distinct advantages over the threonine pathway: i) it involves only three enzymatic steps; ii) it circumvents the constraints imposed by the negative feedback of threonine synthesis; iii) it does not require ATP and NADPH input, but generates a net production of NADH, effectively channeling carbon flux toward 2KB biosynthesis. Genome‐scale metabolic network models (GEMs) were further used to simulate and compare the two pathways. The simulation results suggested that, under various conditions of substrate consumption and biomass lower bounds, the citramalate pathway consistently showed higher 2MBA synthesis flux compared to the threonine pathway (**Figure** 1b,c). Therefore, the citramalate pathway was selected as the preferred route for constructing the 2MBA biosynthetic pathway.

To construct 2MBA biosynthesis pathway based on citramalate pathway in *E. coli*, we first overexpressed citramalate synthase (CimA) from *Methanococcus jannaschii*, together with the endogenous 3‐isopropylmalate dehydratase (LeuCD) and 3‐isopropylmalate dehydrogenase (LeuB).^[^ ^32^, ^33^ ^]^ The 2MBA biosynthesis was then completed by employing the Ehrlich pathway in 2KB overproducing strain, in which a promiscuous 2‐ketoacid decarboxylase (encoded by *kivD*) and aldehyde dehydrogenase (encoded by *ALD2*) were expressed (Figure 1a). The resulting strain LY15 successfully produced 61.78 ± 5.59 mg L^−1^ 2MBA after 48 h (Figure S1a, Supporting Information). The expression of the citramalate pathway also led to the accumulation of 39.80 ± 10.22 mg L^−1^ of 2KB and 40.09 ± 4.19 mg L^−1^ of 2‐keto‐3‐methylvalerate (KMV), which were key intermediates for 2MBA synthesis (Figure S1a, Supporting Information). Furthermore, we observed a significant accumulation of the byproduct acetate (6.38 ± 0.47 g L^−1^) after fermentation when all glucose was consumed, indicating that only a small fraction of the carbon source was utilized for 2MBA biosynthesis (Figure S1b, Supporting Information).

### 2.2 Systematically Engineering of 2MBA Biosynthesis in *E. coli* Mono‐Culture

To further increase 2MBA production, we systematically engineered the initial strain LY15 from three aspects: i) blocking competitive pathways to improve the precursor pools of PYR and 2KB, ii) overexpressing acetohydroxyacid synthase (AHAS) to enhance KMV supply, and iii) optimizing the pathway expression system to coordinate the genes expression of 2MBA biosynthesis.

#### 2.2.1 Blocking Competitive Pathways to Improve the Precursor Pools

**Figure 2:**
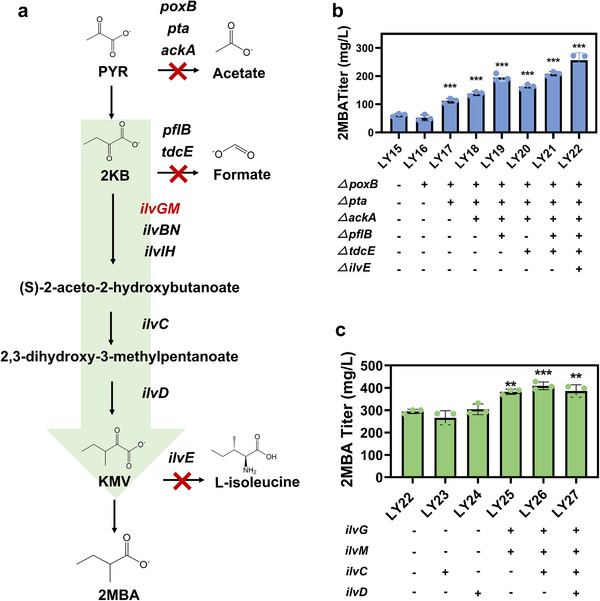
Systematic engineering of 2MBA biosynthesis in *E. coli* mono‐culture. a) Schematic overview of the strategies used to enhance the supply of key intermediates and increase 2MBA production. Red letters indicate the up‐regulated genes *ilvGM*. The red “X” marks indicate pathway deletion via chromosomal gene deletion. b) 2MBA production in relevant strains with competitive genes deletion. c) 2MBA production in strains LY23‐LY27 with *ilvG*, *ilvM*, *ilvC*, and *ilvD* overexpression. Statistical analysis was performed using a student's t‐test (one‐tailed; * *p* < 0.05, ** *p* < 0.01, and *** *p* < 0.001; two‐sample unequal variance). The mean ± s.d. of three biological replicates of a representative measurement is shown.

To increase the PYR precursor pool, we first successively deleted the genes *poxB*, *pta*, and *ackA* associated with acetate synthesis, resulting the strains LY16, LY17, and LY18, respectively (**Figure** **2**a). As expected, deleting these three genes significantly decreased acetate titer from 6.38 ± 0.47 g L^−1^ to 0.25 ± 0.028 g L^−1^ in the strain LY18 (Figure S2a, Supporting Information). Although the growth of the strain LY18 was slightly affected by the deletion of *pta* and *ackA* (Figure S2b, Supporting Information), the titer of 2MBA significantly increased from 61.78 ± 5.59 mg L^−1^ to 137.66 ± 7.21 mg L^−1^ (Figure 2b). Meanwhile, blocking acetate synthesis redirected the metabolic flux to PYR accumulation of 42.47 ± 11.70 mg L^−1^ (Figure S3a, Supporting Information), leading to an improvement in 2KB and KMV synthesis to 403.94 ± 6.012 mg L^−1^ (Figure S3b, Supporting Information) and 34.40 ± 0.97 mg L^−1^ (Figure S3c, Supporting Information), respectively.

Next, we aimed to further increase the supply of intermediates by targeting the degradation pathways of PYR and 2KB. To achieve this, we knocked out the PYR‐formate lyase encoding gene (*pflB*) and the 2KB‐formate lyase encoding gene (*tdcE*) in strain LY18.^[^ ^34^, ^35^, ^36^ ^]^ Although the double knockout strain LY21 did not further enhance PYR and 2KB accumulation, the titer of 2MBA significantly increased to 208.25 ± 7.65 mg L^−1^ (Figure 2b), a 1.5‐fold increase compared with the strain LY18. However, it should be noted that the deletion significantly decreased the KMV titer to 8.59 ± 1.09 mg L^−1^ (Figure S3c, Supporting Information), indicating insufficient KMV supply for 2MBA synthesis.

Finally, the gene *ilvE* was deleted in the strain LY21, which encoded a branched‐chain amino acid transaminase, to prevent the conversion of KMV into isoleucine (strain LY22) (Figure 2a). After the gene *ilvE* deletion, the concentration of KMV increased to 21.45 ± 0.79 mg L^−1^ (Figure S3c, Supporting Information). Consequently, the titer of 2MBA increased to 256.69 ± 26.26 mg L^−1^, which 4.2 times higher than that of the strain LY15 (Figure 2b). Although the deletion of the above genes has significantly improved the 2MBA production, the accumulation of 2KB demonstrated that the expression level of the branched‐chain amino acid biosynthesis module in strain LY22 alone was not sufficient.

#### 2.2.2 Overexpressing Acetohydroxyacid Synthase (AHAS) to Enhance KMV Supply

The biosynthesis of KMV from 2KB involves a series of enzymatic reactions. Initially, acetohydroxyacid synthase (AHAS) catalyzes the condensation of 2KB and PYR in the production of (S)‐2‐aceto‐2‐hydroxybutanoate and the release of CO_2_. Subsequently, ketol‐acid reductoisomerase, encoded by *ilvC*, utilizes one molecule of NADPH to convert (S)‐2‐aceto‐2‐hydroxybutanoate into (R)‐2,3‐dihydroxy‐3‐methylpentanoate. Finally, dihydroxy‐acid dehydratase, encoded by *ilvD*, facilitates the conversion of (R)‐2,3‐dihydroxy‐3‐methylpentanoate to KMV (Figure 2a).

Notably, *E. coli* BL21 possesses three distinct AHAS isozymes: AHAS I encoded by *ilvBN*, AHAS II encoded by *ilvGM* (inactive in *E. coli* K‐series), and AHAS III encoded by *ilvIH*. Previous studies have indicated that AHAS II demonstrates superior performance compared to the other AHAS isozymes AHAS in converting 2KB to 2‐methyl‐1‐butanol, highlighting a better substrate affinity of AHAS II toward 2KB. Furthermore, AHAS II has displayed resistance to L‐valine.^[^ ^37^, ^38^ ^]^ Furthermore, inactivate of these three enzymes in *E. coli* BL21 showed that the AHAS II (*ilvGM*) had the detrimental effect on KMV synthesis (Figure S4, Supporting Information).

Therefore, to drive the 2KB pool toward KMV, we gradually overexpressed the genes of *ilvGM, ilvC*, and *ilvD*, which are related to the branched‐chain ketoacid synthesis module, resulting strains LY23‐LY27. Among these, strain LY25, with only *ilvGM* overexpression, showed a significant increase in 2MBA production, reaching 382.28 ± 11.53 mg L^−1^, which was a 48.9% increase compared to the strain LY22 (Figure 2c). However, further overexpression of *ilvC* and *ilvD* in the strain LY25 sustained a high level of 2MBA production, but did not show significantly increase compared to strain LY25 (Figure 2c; Figure S5, Supporting Information).

#### 2.2.3 Optimizing the Expression System to Coordinate the Genes Expression

**Figure 3:**
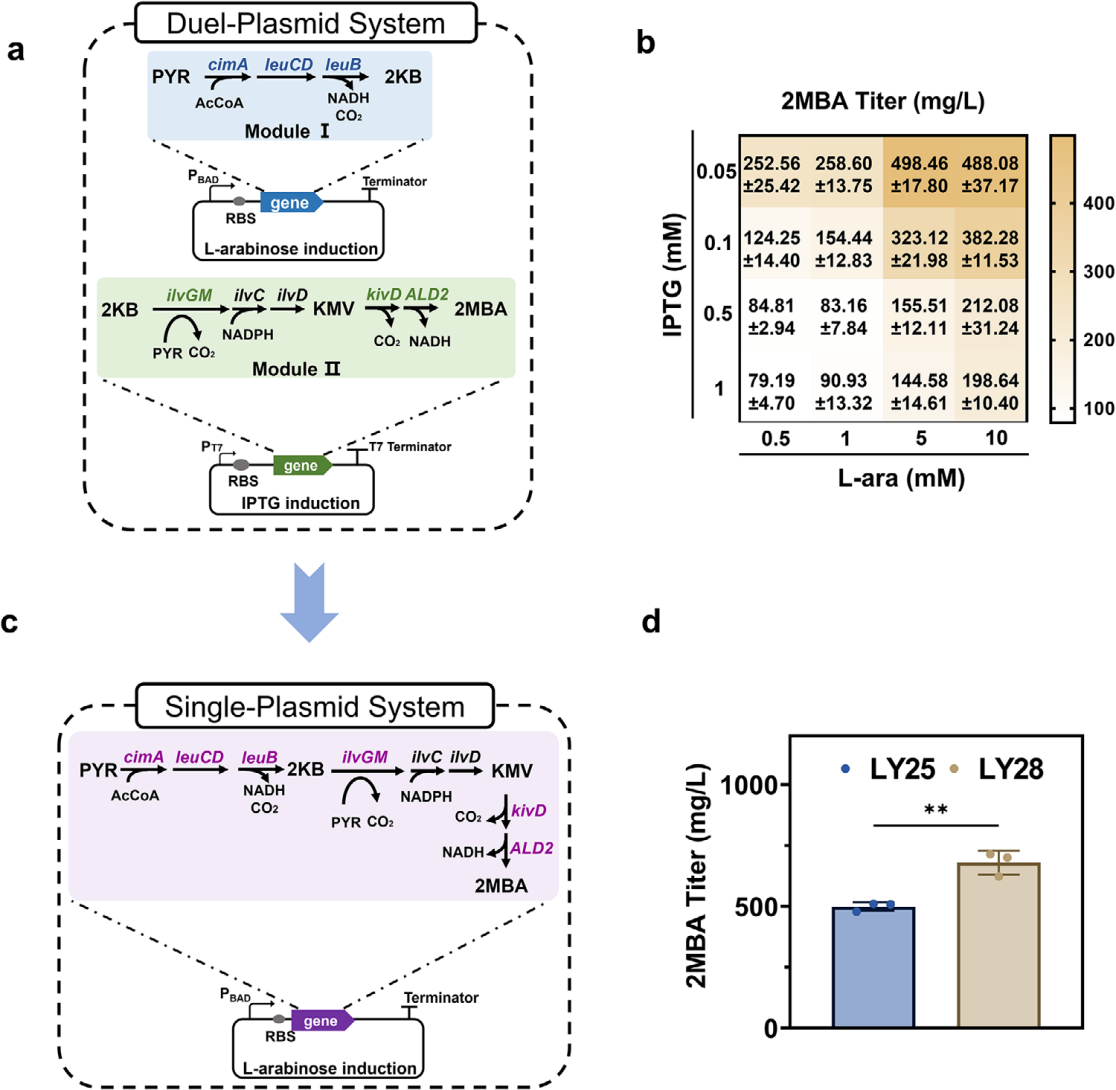
Optimizing the expression system to coordinate the genes expression for 2MBA biosynthesis. a) Schematic overview of the dual‐plasmid system in strain LY25. b) 2MBA titer in orthogonal experiments testing various concentrations of L‐arabinose and IPTG. c) Schematic overview of the single‐plasmid system in strain LY28. d) 2MBA titer in the mono‐culture of strains LY25 and LY28. Statistical analysis was performed using a student's t‐test (one‐tailed; * *p* < 0.05, ** *p* < 0.01, and *** *p* < 0.001; two‐sample unequal variance). The mean ± s.d. of three biological replicates of a representative measurement is shown.

Gene overexpression can lead to an increased metabolic burden on host cells, which may result in the accumulation of byproducts and hinder the efficient synthesis of the target product. Initially, we employed a dual‐plasmid system to express the genes involved in 2MBA synthesis. Specifically, the genes responsible for the conversion of PYR to 2KB were constructed in an L‐arabinose inducible high‐copy plasmid pE8a (Module I, **Figure** **3**a), while the genes for the further conversion of 2KB to 2MBA were placed on an IPTG inducible plasmid pACYC‐Duet‐1 (Module II, **Figure** 3a). To optimize these two modules, we performed a series of orthogonal experiments testing various concentrations of L‐arabinose and IPTG. Under the influence of different inducer combinations, the synthesis of key precursors 2KB and KMV showed significant differences (Figure S6, Supporting Information), which in turn affects 2MBA titer. As shown in Figure 3b, the highest titer of 498.46 ± 17.80 mg L^−1^ 2MBA was achieved with 0.05 mM IPTG and 5 mM L‐arabinose, a 30.39% increase compared to the initial conditions. However, a notable decrease in cell growth was observed, which might be attributed to the metabolic stress of the dual‐plasmid system.

To alleviate this stress, we integrated all the genes into one plasmid pE8a, creating strain LY28 (Figure 3c). The results revealed that, compared to the strain LY24 containing two plasmids, strain LY28 exhibited a faster growth rate during the 2MBA biosynthesis, reaching the stationary phase ≈12 h earlier than LY24 (Figure S7a, Supporting Information). This accelerated growth also led to an increased glucose consumption rate of the LY28 strain (Figure S7b, Supporting Information). When both 2KB and KMV were at low accumulation levels (Figure S7c,d, Supporting Information), expression system optimization enabled the strain LY28 produced 678.78 ± 49.04 mg L^−1^ 2MBA (Figure 3d), a 36.18% higher titer than the dual‐plasmid system of strain LY25. Consequently, the engineered strain *E. coli* LY28 exhibited a 2MBA titer and yield of 678.78 ±·49.04 mg L^−1^ and 0.034 g/g glucose, respectively.

### 2.3 Predicting Key Nodes and Division of Labor in *E. coli* Consortia by CulECpy Model

Compared to mono‐culture system, synthetic consortia enable more flexible and efficient 2MBA biosynthesis by rationally allocating pathways across different hosts, effectively reducing cross‐reactivity and metabolic burden. Nevertheless, one of the main challenges lies in determining the optimal functional distribution and pathway allocation among members of the consortium. Conventional experimental approaches often require substantial time and resources, whereas computational pathway allocation simulations offer a cost‐effective alternative for strategy optimization. To this end, we employed our previously developed Co‐Cultivated Enzyme‐Constrained Metabolic Network Model (CulECpy).^[^ ^31^ ^]^

**Figure 4:**
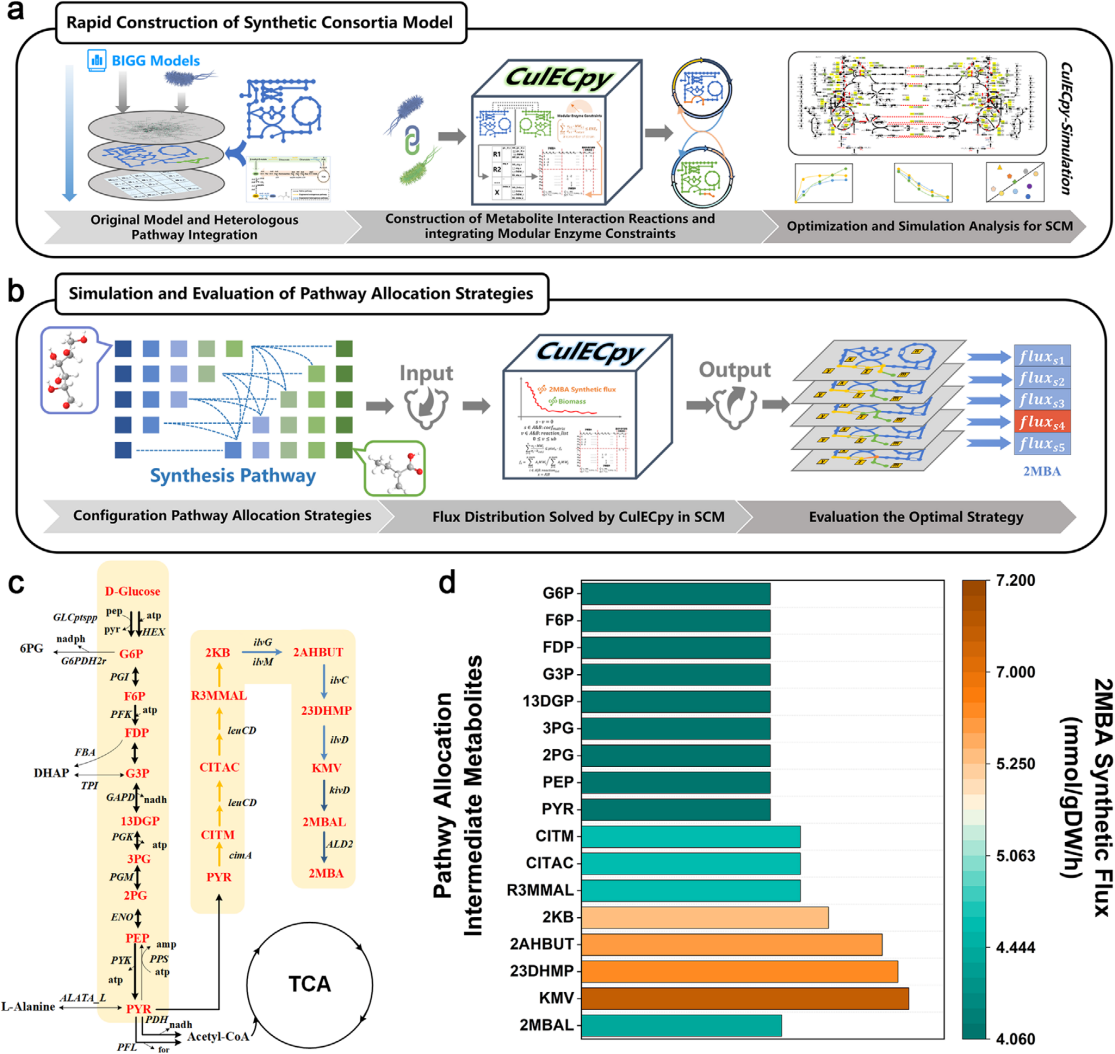
Rational construction of 2MBA synthetic consortia guided by CulECpy. a) Rapid construction of synthetic consortia models. b) Simulation and evaluation of pathway allocation strategies. c) Schematic diagram of the complete synthetic pathway of 2MBA from glucose. d) Rational analysis using CulECpy to assess differences in 2MBA synthesis with 17 potential intermediate metabolites as pathway allocation nodes. (G6P: D‐Glucose 6‐phosphate, F6P: D‐Fructose 6‐phosphate, FDP: D‐Fructose 1,6‐bisphosphate, G3P: Glyceraldehyde 3‐phosphate, 13DGP: 3‐Phospho‐D‐glyceroyl phosphate, 3PG: 3‐Phospho‐D‐glycerate, 2PG: D‐Glycerate 2‐phosphate, PEP: Phosphoenolpyruvate, PYR: Pyruvate, CITM: Citramalate, CITAC: Citraconate, R3MMAl: β‐methyl‐D‐malate, 2KB: 2‐ketobutyrate, 2AHBUT: 2‐aceto‐2‐hydroxy‐butyrate 2,3‐dihydroxy‐3‐methylvalerate, 23DHMP: 2,3‐Dihydroxy‐3‐methylpentanoate, KMV: 2‐keto‐3‐methyl‐valerate, 2MBAL: 2‐methylbutanal, 2MBA: 2‐methylbutyric acid).

The CulECpy facilitates the simultaneous evaluation of biomass and 2MBA biosynthesis through a multi‐objective algorithm, enabling predictions of synthesis capacity variations under different pathway allocation strategies. As illustrated in **Figure** **4**a,b, we initially selected the original *E. coli* GEM, iML1515, as the foundation constructing the 2MBA co‐culture synthesis model. This original model was then modified to incorporate the 2MBA biosynthetic pathway by embedding heterologous reactions, with reaction information sourced from the BioCyc database. To achieve flux connection between different strains in a consortium, the built‐in script of CulECpy was used to establish external metabolite interactions, linking 359 metabolite pairs. Enzyme kinetic parameters for *E. coli* were retrieved from enzyme databases, and modular enzyme constraints provided by CulECpy were integrated into the model, resulting in a comprehensive synthetic consortium model (SCM). This SCM, comprising 4123 metabolites and 6180 reactions, was converted into a Pyomo matrix format using CulECpy. Multi‐objective functions for biomass and 2MBA synthesis were then defined, followed by global flux balance analysis. By inputting different pathway allocation strategies into CulECpy, we computed the corresponding global flux distributions, using the optimal 2MBA synthetic flux under basic growth conditions as the evaluation criterion.

Building on this framework, we next examined the complete biosynthetic pathway in *E. coli*, from direct glucose utilization to final 2MBA synthesis, encompassing 17 potential intermediate metabolites (**Figure** **4c**; Table S5, Supporting Information). Each metabolite represents a potential node for pathway allocation within the consortium. By systematically evaluating these 17 sets of intermediate metabolites as potential nodes for pathway allocation and comparing their 2MBA synthesis flux, we identified the most effective intermediate 2‐keto‐3‐methylvalerate (KMV), which has the highest 2MBA synthetic flux of 7.037 mmol/gDW/h among other intermediate metabolites (Figure **4d**).

**Figure 5:**
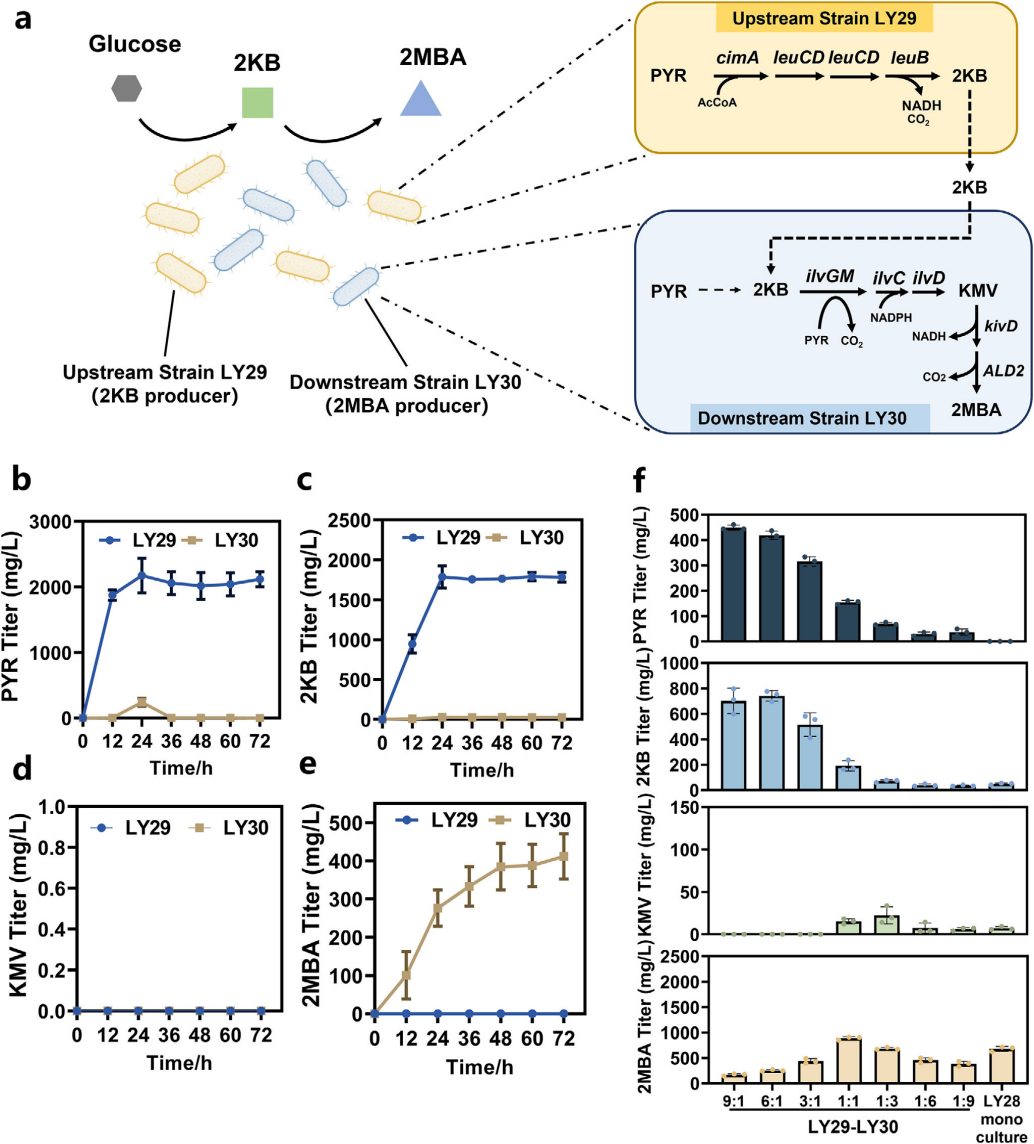
Consortium I with 2KB as single node for 2MBA biosynthesis. a) Schematic of *E. coli – E. coli* consortium composed of two strains LY29 and LY30 for 2MBA production. Upstream strain LY29, with *poxB*, *pta*, *ackA*, *ilvIH*, *ilvGM*, *ilvBN* deletion and *cimA*, *leuB*, *leuC*, and *leuD* overexpression, was able to biosynthesize excess 2KB, providing precursors for downstream strain LY30 to biosynthesize KMV and 2MBA. Downstream strain LY30, with *poxB*, *pta*, *ackA*, *pflB*, *tdcE*, *ilvE* deletion and *ilvGM*, *kivD*, and *ALD2* overexpression, specialized in KMV and 2MBA biosynthesis. b‐e) Functional validation of upstream strain LY29 and downstream strain LY30. Temporal profiles of (b) PYR, (c) 2KB, (d) KMV, and (e) 2MBA biosynthesis during the mono‐culture of strain LY29 and strain LY30. f) Comparison of PYR, 2KB, KMV, and 2MBA titers in LY28 mono‐culture and LY29‐LY30 co‐culture. The data are presented as the average of three independent experiments, and error bars indicate standard errors.

### 2.4 Rational Construction of *E. coli* Consortia for Enhanced 2MBA Production

In addition to the KMV node that was predicted by the CulECpy model, the 2KB node was also manually selected for comparison, as it is the key intermediate linking the two pathway modules and exhibits a large fluctuation depending on different metabolic strategies. Furthermore, the two nodes with overlapping functions were also tested. Based on these considerations, three distinct consortia were designed:

Consortium I: 2KB as single node, with the upstream strain specializing in 2KB biosynthesis and the downstream strain possessing the ability of KMV and 2MBA biosynthesis;
Consortium II: KMV as single node, with the upstream strain specializing in KMV biosynthesis and the downstream strain possessing the ability of 2MBA biosynthesis;
Consortium III: 2KB and KMV as double‐node, simultaneously enhancing the transformation from 2KB to KMV in the upstream and downstream strains with pathway overlapping.

#### 2.4.1 Consortium I: 2KB as Single Node

To construct the Consortium I with 2KB as a single node, the upstream strain LY29 was designed to overproduce 2KB, while the downstream strain LY30 converted 2KB into KMV and ultimately 2MBA (Figure 5a). The individual functions of LY29 and LY30 were evaluated to validate their roles within Consortium I. Strain LY29 effectively synthesized 2117.82 ± 113.92 mg L^−1^ PYR (Figure 5b) and 1781.99 ± 59.97 mg L^−1^ 2KB (Figure 5c) from glucose, demonstrating its effectiveness in supplying 2KB for downstream processes. In contrast, the downstream strain LY30 did not produce significant amounts of PYR or 2KB but accumulated 2MBA at 411.67 ± 59.18 mg L^−1^ (Figure 5e), confirming its capability to utilize 2KB for 2MBA synthesis. The absence of KMV accumulation in both strains can be explained by the inhibition of KMV synthesis in strain LY29 and its conversion to 2MBA in strain LY30 (Figure 5d).

After confirming the capabilities of strains LY29 and LY30 to synthesize 2KB and 2MBA, respectively, Consortium I was assembled and optimized by adjusting the inoculation ratios of the upstream and downstream strains. As shown in **Figure5f,** significant accumulations of PYR and 2KB were observed at strain ratios of LY29:LY30 ranging from 9:1 to 3:1, with no detectable KMV accumulation under these conditions. Further increase the proportion of strain LY30 resulted in a remarkable decrease in the concentrations of PYR and 2KB. However, the KMV concentration did not showed a corresponding increase, with a maximum level of only 22.40±10.08 mg L^−1^ at ratio of 1:3, which may be attributed to the inadequate supply of 2KB.

**Figure 6:**
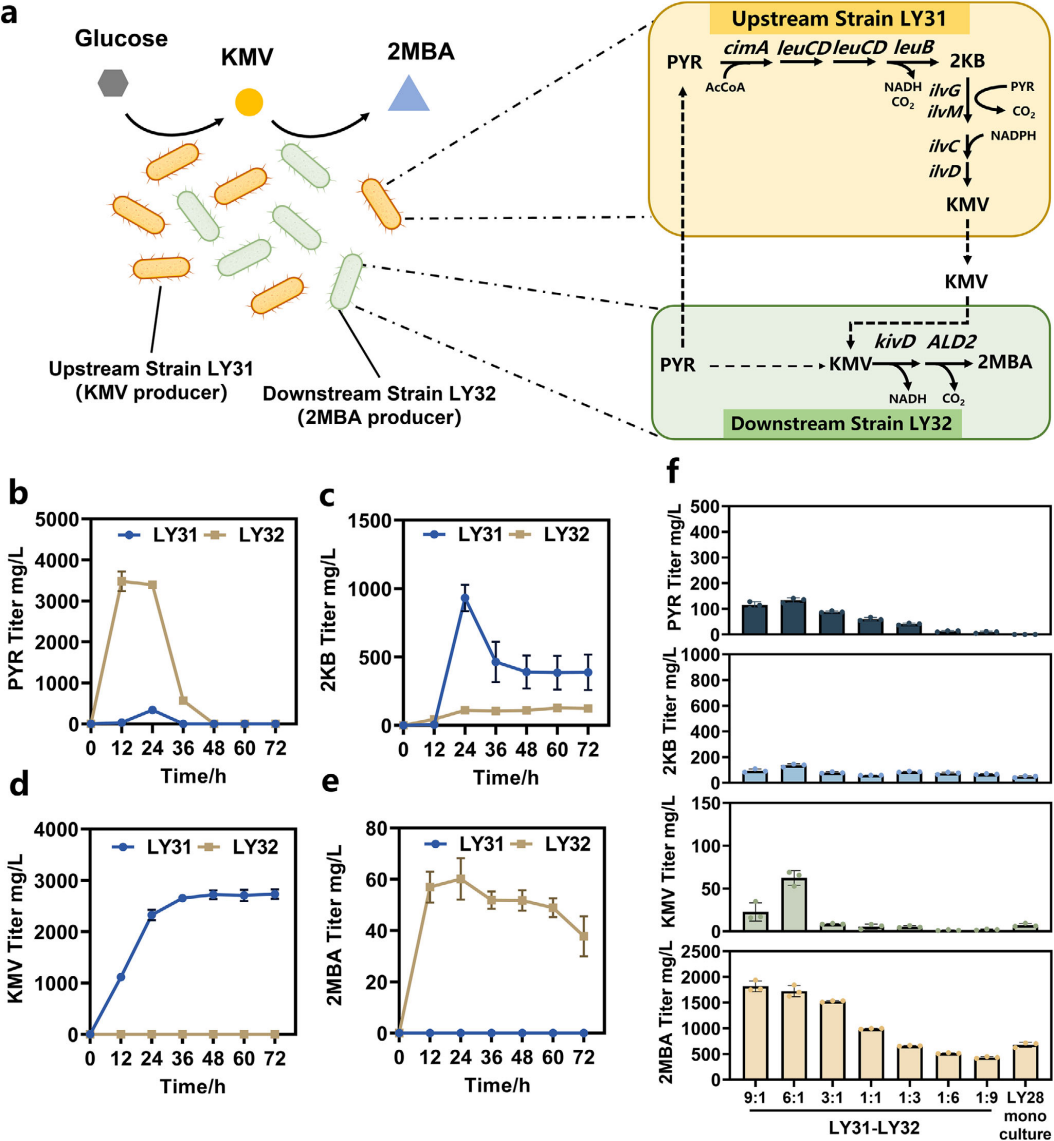
Consortium II with KMV as single node for 2MBA biosynthesis. a) Schematic of *E. coli – E. coli* consortium composed of two strains LY31 and LY32 for 2MBA production. Upstream strain LY31 with the deletions of *poxB*, *pta*, *ackA*, *pflB*, *tdcE*, *ilvE* and the overexpression of *cimA*, *leuB*, *leuC*, *leuD*, *ilvGM*, was able to biosynthesize excess KMV and providing precursors for downstream strain LY32 to biosynthesize 2MBA. Downstream strain LY32 with a similar set of deletions (*poxB*, *pta*, *ackA*, *pflB*, *tdcE*, and *ilvE*) to LY31 and the overexpression of *kivD* and *ALD2*, specialized in 2MBA biosynthesis. b‐e) Functional validation of upstream strain LY31 and downstream strain LY32. Temporal profiles of (b) PYR, (c) 2KB, (d) KMV, and (e) 2MBA biosynthesis during the mono‐culture of strain LY31 and strain LY32. f) Comparison of PYR, 2KB, KMV, and 2MBA titers in LY28 mono‐culture and LY31‐LY32 co‐culture. The data are presented as the average of three independent experiments, and error bars indicate standard errors.

The best 2MBA biosynthesis was achieved at an inoculation ratio of 1:1, reaching a 2MBA titer and yield of 887.63 ± 31.61 mg L^−1^ and 0.044 g/g glucose, which represented a 30.77% and 29.41% respectively increase compared to strain LY28 mono‐culture. In contrast, either increasing or decreasing the proportion of the downstream strain LY30 can led to lower 2MBA titer, highlighting the importance of achieving a balanced metabolic exchange between the strains, which was essential for efficient consortium performance.

#### 2.4.2 Consortium II: KMV as Single Node

We next engineered Consortium II with KMV as the single node, which was predicted to be the most effective intermediate for pathway allocation. The upstream strain LY31 was designed to provide sufficient PYR for 2KB synthesis and further facilitated the conversion of 2KB to KMV, while the downstream strain LY32 was allowed to efficiently convert from KMV to 2MBA (Figure 6a).

Individual cultivation tests suggested that the upstream strain LY31 demonstrated significant 2KB production of 931.54 ± 96.16 mg L^−1^ at 24 h, followed by a decline to 388.56 ± 129.87 mg L^−1^ due to its conversion into KMV (Figure 6b,c). Notably, strain LY31 exhibited a high capacity for KMV accumulation, reaching up to 2733.22 ± 91.63 mg L^−1^ (Figure 6d). In contrast, LY32 synthesized minimal amounts of KMV, indicating that it primarily consumes KMV for downstream conversion. For 2MBA production, LY32 achieved a detectable concentration of 37.77 ± 7.81 mg L^−1^, while no 2MBA was observed in LY31 cultures (Figure 6e).

Consortium II was constructed with the ability of LY31 to produce KMV and LY32 to convert KMV to 2MBA. As shown in Figure 6f, the overall accumulation of key intermediates, including PYR and 2KB, was significantly lower compared to Consortium I, indicating a more efficient metabolic flux transfer between the strains. The highest concentrations of 2KB and KMV in Consortium II were achieved at inoculation ratio of 6:1, with concentrations of 136.01 ± 12.27 mg L^−1^ and 62.40 ± 8.51 mg L^−1^, respectively. Interestingly, the accumulation of 2KB was independent of the inoculation ratio of the upstream and downstream strains, indicating that the synthesis of 2KB and its conversion to KMV within a single cell was beneficial for enhancing the conversion efficiency of 2KB to KMV.

The final 2MBA titer was strongly influenced by the inoculation ratio of LY31 to LY32. Interestingly, higher inoculations of strain LY32 led to reduced 2MBA productions, likely due to imbalances in metabolic flow and insufficient supply of KMV. The optimal inoculation ratio was 9:1, at which the 2MBA titer and yield reached their peaks at 1817.03 ± 103.73 mg L^−1^ and 0.091 g/g, respectively. These values were 2.67‐fold and 1.07‐fold higher than the maximum titer and yield obtained in the LY28 mono‐culture.

#### 2.4.3 Consortium III: 2KB and KMV as Double‐Node

**Figure 7:**
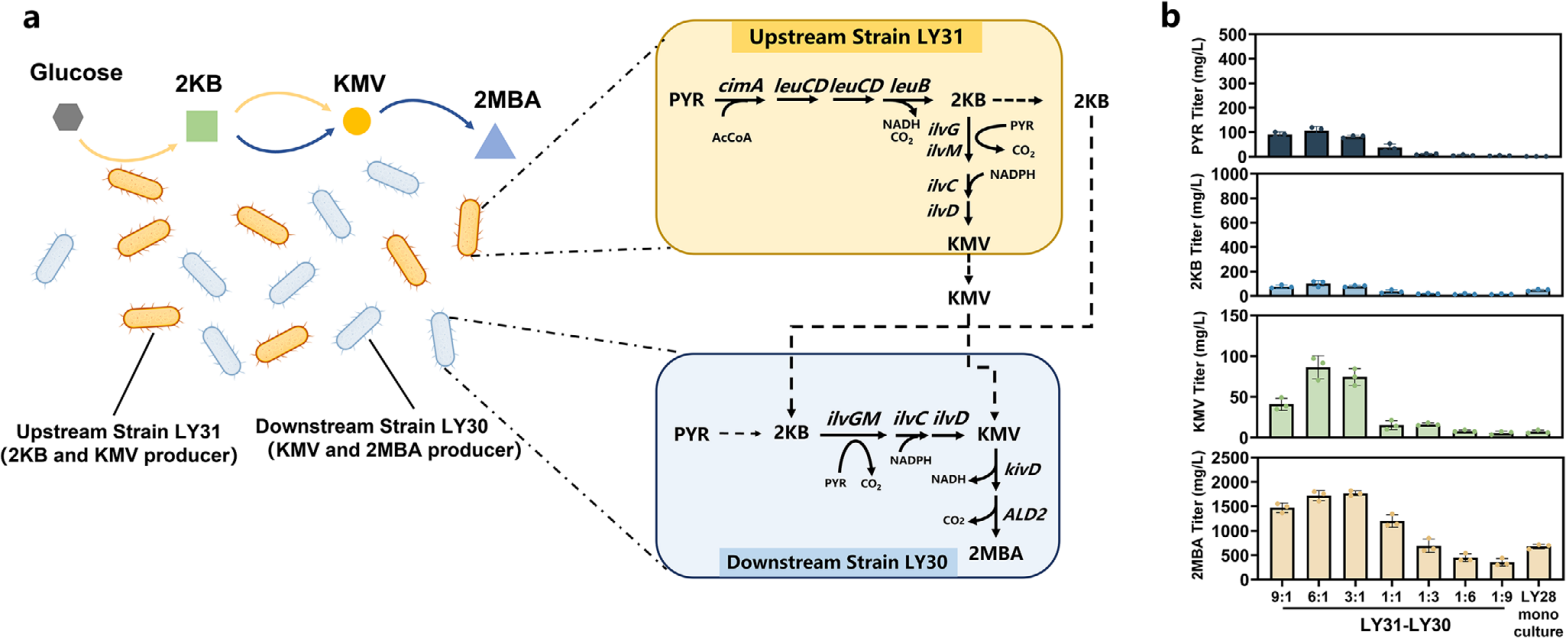
Consortium III with 2KB and KMV as dual‐node for 2MBA biosynthesis. a) Schematic of *E. coli – E. coli* consortium composed of two strains LY31 and LY30 for 2MBA production. Upstream strain LY31 was able to biosynthesize 2KB and KMV, providing precursors for downstream strain LY30 to biosynthesize KMV and 2MBA. Downstream strain LY30, specialized in KMV and 2MBA biosynthesis. b) Comparison of PYR, 2KB, KMV, and 2MBA titers in LY28 mono‐culture and LY31‐LY30 co‐culture. The data are presented as the average of three independent experiments, and error bars indicate standard errors.

Consortium III was designed as a dual‐node system, utilizing both 2KB and KMV as key intermediates. This consortium incorporated the upstream strain LY31 from Consortium II and the downstream strain LY30 from Consortium I, with the genes *ilvGM* overexpressed in both strains (**Figure** **7**a). In consortium III, the primary role of the upstream strain LY31 was to supply sufficient quantities of 2KB and KMV, while the downstream strain LY30 was responsible for converting these intermediates into 2MBA. By enhancing the conversion from 2KB to KMV in both strains, Consortium III had the potential to achieve higher 2MBA titers compared to Consortia I and II. Additionally, the surplus PYR produced by LY30 could serve as a substrate for LY31, creating a dynamic and interdependent metabolic relationship between the two strains.

As depicted in Figure 7b, the accumulation of PYR and 2KB in consortium III was similarly influenced by the inoculation ratio of LY31 to LY30. The highest levels of PYR and 2KB were observed at an inoculation ratio of 6:1. However, further increases in strain LY30 inoculation led to reduced accumulation of these intermediates. The accumulation of KMV demonstrated significant variability with changes in the inoculation ratio. At the 6:1 ratio, KMV reached its highest accumulation of 86.38 ± 14.10 mg L^−1^, representing a 38.41% increase compared to Consortium II. This increase validated the enhanced expression of the branched‐chain ketoacid synthesis pathway in Consortium III, enabled by the overexpression of *ilvGM* in both strains.

Furthermore, 2MBA titer increased as the inoculation ratio of LY30 to LY31 shifted from 9:1 to 3:1. However, further increases in strain LY30led to a decline in 2MBA titer. The highest titer and yield of 2MBA, 1766.60 ± 50.41 mg L^−1^ and 0.088 g/g, were achieved at a 3:1 inoculation ratio, respectively. These figures represented a 2.60‐fold increase in titer and a 1‐fold increase in yield compared to the maximum concentrations observed in the LY28 mono‐culture.

## 3 Discussion

In this study, we successfully developed a novel citramalate‐derived pathway for 2‐methylbutyric acid (2MBA) biosynthesis, offering several key advantages over the threonine‐derived pathway. First, it avoids the negative feedback inhibition caused by excessive threonine synthesis,^[^ ^39^, ^40^ ^]^ which can limit production efficiency. Second, it simplifies the biosynthetic process by reducing the number of steps, cutting three steps compared to the threonine‐based pathway.^[^ ^16^, ^41^ ^]^ Thirdly, the final decarboxylation step catalyzed by LeuB potentially served as an irreversible driving force, directing carbon flux toward the synthesis of 2‐ketobutyrate (2KB).^[^ ^32^ ^]^ These advantages made the citramalate‐derived pathway a promising alternative for efficient and streamlined 2MBA production.

Initially, we constructed the citramalate pathway in *E. coli* mono‐culture, resulting in a 2MBA production of 61.78 ± 5.59 mg L^−1^. Through comprehensive engineering of the entire pathway, including blocking competitive pathways, overexpressing acetohydroxyacid synthase (AHAS), and optimizing the pathway expression system, we were able to significantly increase the 2MBA titer to 678.78 ± 49.04 mg L^−1^, a 10.9‐fold increase compared with the initial strain.

While mono‐culture systems provide a foundational platform, synthetic consortia offer distinct advantages for multi‐step biosynthesis through spatial and functional specialization. The division of labor across engineered strains reduces individual metabolic burden while enabling modular optimization via strain ratio adjustments.^[^ ^42^, ^43^, ^44^ ^]^ However, the biosynthesis pathway for 2MBA involves multiple intermediates and complex biochemical reactions. Coordinating these pathways within a synthetic consortium, while ensuring efficient conversion of intermediates, presents a great challenge in terms of pathway optimization and metabolic flux control.

Traditionally, constructing a synthetic consortium for efficient metabolic processes typically involved complex and time‐consuming trial‐and‐error methods to distribute metabolic tasks among different strains. To greatly enhance the efficiency of constructing such consortia by providing a more rational and accurate method for pathway allocation, Co‐Cultivated Enzyme Constraint Metabolic Network Model (CulECpy) was used.^[^ ^23^, ^26^, ^31^ ^]^ In the entire pathway for synthesizing 2MBA from glucose, we systematically evaluated 17 major intermediate metabolites as pathway allocation nodes, with two key nodes emerging: 2KB and 2‐keto‐3‐methylvalerate (KMV). We subsequently implemented three distinct pathway allocation strategies, either independently or in combination, for each of the two potential intermediate metabolites, thereby generating three distinct *E. coli* – *E. coli* synthetic consortia: Consortium I with 2KB as single node, Consortium II with KMV as single node, and Consortium III with 2KB and KMV as double‐node.

The division of labor for converting 2KB to KMV was found to significantly influence 2MBA biosynthesis in the consortia. In Consortium I, where 2KB was used as a single node, the conversion of 2KB to KMV was overexpressed in the downstream strain LY30. This physical separation of the pathway resulted in the upstream strain LY29 synthesizing excessive 2KB, leading to high 2KB concentrations in both upstream and downstream strains during fermentation (Figure 5c). Excessive 2KB accumulation was reported to be toxic to the cells,^[^ ^32^, ^45^, ^46^, ^47^ ^]^ potentially limiting 2MBA production. Therefore, Consortium I, which used 2KB as a single node, performed the worst in the biosynthesis of 2MBA, with the highest yield being only 887.63 ± 31.61 mg L^−1^ at an initial ratio of LY29:LY30 = 1:1 (Figure 5f). Differently, in Consortium II, the conversion of PYR to 2KB and subsequently 2KB to KMV both occurred within the upstream strain LY31, eliminating the physical separation and facilitating the transformation of 2KB to KMV. This reduction in the accumulation of 2KB also served as a detoxification effect and resulted in the highest 2MBA titer of 1817.03 ± 103.73 mg L^−1^ and yield of 0.091 g/g glucose (Figure 6f).

However, it should be noticed that pathway allocation with overlapping functions can also affect the accumulation of key intermediates and the timely production of 2MBA. In Consortium III, where the 2KB‐to‐KMV module was overexpressed in both the upstream and downstream strains, KMV accumulation increased compared to Consortium II (Figure 7b), though 2MBA production did not significantly improve. The optimal inoculation ratio shifted from 9:1 to 3:1, indicating that strengthening the 2KB‐to‐KMV pathway in the downstream strains might reduce the biosynthetic capacity of KMV‐to‐2MBA (Figures 6f and 7b). This result suggested that the overexpression of 2KB‐to‐KMV conversion in the downstream strains might have competed for resources required for 2MBA biosynthesis from KMV.

The dynamic balance of microbial community structure in consortium plays a crucial role in 2MBA biosynthesis. Specifically, in Consortium II, the proportion of upstream strains continuously decreased, while the downstream strains gradually became dominant over time. This dynamic change may be beneficial for improving overall 2MBA production. In the early stages, upstream strains rapidly consume glucose to synthesize the key precursor KMV. In the later stages, downstream strains utilize this precursor to further produce 2MBA (Figure S8, Supporting Information). This division of labor enhances 2MBA biosynthesis efficiency by ensuring a steady supply of precursors and optimizing metabolic flow within the consortium. However, developing community control strategies, such as auxotrophic relationships or dynamic control modules, could potentially help build a more stable system and further enhance synthesis efficiency.

In conclusion, we successfully achieved the bioproduction of 1817.03 ± 103.73 mg L^−1^ 2MBA using a rationally designed *E. coli – E. coli* consortium, which was 28‐fold higher than the original mono‐culture strain developed in this study. This achievement marks a significant advancement in the application of synthetic consortium with the use of advanced models for rational design. Our work not only advances the field of synthetic biology but also offers valuable insights into the development of more efficient and sustainable biosynthetic processes. With data‐driven and optimized models, this work paves the way for more efficient and sustainable biosynthesis processes in the future.

## 4 Experimental Section

### Media

Luria‐Bertani (LB) medium was used for cultivation of seed cultures. LB medium comprised the following (per liter): 10 g tryptone, 5 g yeast extract, and 10 g NaCl. M9Y medium was used for 2MBA biosynthesis in *E. coli*. One liter of M9Y medium contained 20 g glucose, 11.3 g M9 mixed salts, 5 g yeast extract, 15.7 g 3‐morpholinopropanesulfoinc acid, 2 mM MgSO_4_, 0.1 mM CaCl_2_, 2.78 mg FeSO_4_, 1 mg Vitamin B1, 1 mL trace elements. The working concentrations of trace elements were: 0.371 mg L^−1^ (NH_4_)_6_Mo_7_O_24_·4H_2_O, 0.243 mg L^−1^ H_3_BO_3_, 0.288 mg L^−1^ ZnSO_4_, 0.714 mg L^−1^ CoCl_2_ 0.374 mg L^−1^ CuSO_4_·5H_2_O, 1.583 mg L^−1^ MnCl_2_. L‐arabinose and IPTG were added to medium at final concentrations of 10 mM and 0.1 mM, respectively. When needed, antibiotics were supplemented into the medium to the following final concentrations: 100 µg mL^−1^ ampicillin, 25 µg mL^−1^ chloramphenicol.

### Culture Conditions

For the mono‐culture, seed cultures were first cultivated overnight at 37 °C in LB medium with ampicillin and chloramphenicol. After overnight growth, seed cultures were inoculated at a 1% v/v into 250 mL Erlenmeyer flasks containing 25 ml M9Y medium with antibiotics. Fermentation was carried on at 37 °C and 200 rpm. When the OD600 of cultivation reached 0.4–0.6, inducers were added. After 72 h of cultivation, samples were taken for HPLC analysis. When needed, antibiotics were supplemented into the medium to the following final concentrations: 100 µg mL^−1^ ampicillin, 25 µg mL^−1^ chloramphenicol.

For the co‐culture experiment, seed cultures of the upstream and downstream strains were individually cultivated overnight at 37 °C in LB medium supplemented with 100 µg mL^−1^ ampicillin. After 12 h of growth, the cell density (OD600) of each strain was measured. Once the OD600 values of the activated upstream and downstream strains were confirmed to be consistent, the initial inoculation volumes for each strain were calculated based on the desired inoculation ratio. The strains were then inoculated into 250 mL Erlenmeyer flasks containing 25 mL of M9Y medium with 100 µg mL^−1^ ampicillin at a final inoculation volume of 1% (v/v). Fermentation was conducted at 37 °C with shaking at 200 rpm. When the OD600 of the fermentation broth reached 0.4‐0.6, L‐arabinose was added to induce expression. After 72 h of cultivation, samples were collected for HPLC analysis.

### Strains and Plasmid Construction

Custom DNA oligonucleotide primers were synthesized by GENEWIZ, Inc (Table S1, Supporting Information). The plasmids and strains used in this study are listed in Tables S2 and S3 (Supporting Information), respectively. *E. coli* trans10 (TransGen Biotech Co., Ltd., Beijing, China) was adopted for cloning and plasmid propagation. *E. coli* BL21 (DE3) (TransGen Biotech Co., Ltd., Beijing, China) was used as a host for metabolic engineering. TransStart FastPFU DNA polymerase (TransGen Biotech Co., Ltd., Beijing, China) was used for PCR amplification of target DNA fragments as well as vector linearization. DNA gel purification, plasmid extraction kits and Gibson Assembly Cloning Kit was purchased from Vazyme (Vazyme Biotech Co., Ltd, Nanjing, China). All heterologous genes (*cimA*, *kivD*, and *ALD2*) codon optimized were synthesized by GENEWIZ, Inc (Table S4, Supporting Information). The Gibson Assembly Cloning Kit was used for fragment assembly to obtain plasmids.

### Deletion of Chromosomal Genes

The plasmids used to delete chromosomal genes are listed in Table S3 (Supporting Information). The CRISPR/Cas9 system was used for rapid genes deletion.^[^ ^48^ ^]^ The plasmid pEcCas was introduced into the parental strain and incubated overnight in the LB medium with kanamycin. 10 mM L‐arabinose was added to the system for λ‐ Red and pEcΔ was transferred. After recovery culture, the culture medium was seeded to an LB agar plate containing 50 mg L^−1^ of kanamycin and 100 mg L^−1^ of spectinomycin. The target gene deletion was confirmed by colony direct PCR. The plasmids pEcCas, and pEcΔ was eliminated from bacterial the target‐gene‐deficient strain. All fragments inserted in the plasmids used to inactivate the respective genes were amplified using direct colony PCR using the *E. coli* BL21 (DE3) genomic DNA as a template.

### Determination of the Strain‐to‐Strain Ratio

The strain‐to‐strain ratio of the two‐strain co‐culture was analyzed by an antibiotic selection method. In the co‐culture system, the upstream strain contained a plasmid with resistance genes of ampicillin and kanamycin, while the downstream strain contained a plasmid with resistance genes of ampicillin and spectinomycin. 10 µL of the co‐culture sample was diluted by a factor of 10^5^–10^6^ before being spread onto an LB agar plate containing kanamycin and spectinomycin, respectively. After incubation for 24 h, the upstream strain carrying the kanamycin resistance gene grew on the plate with kanamycin, and the downstream strain carrying the spectinomycin resistance gene grew on the plate with spectinomycin. The number of colonies on the plates with different resistances was counted separately to calculate their proportions in the co‐culture population.

### Analytical Methods

Cell growth was determined by measuring OD600 using a UV spectrophotometer. Samples were prepared by centrifuged first at 12 000 rpm for 1 min and the supernatants were filtered with 0.22 µm film. High Performance Liquid Chromatography (Agilent Technologies Inc., California, USA) with the Aminex HPX‐87H column (Bio‐Rad Inc., CA, USA) were used for substances identification and quantification in the fermentation broths, which was employed and operated at 60 °C with 5 mM sulfuric acid as a mobile phase at a flow rate of 0.6 mL min^−1^. Glucose, 2MBA were examined using a refractive index detector. Acetate, pyruvate, 2‐ketobutyrate and 2‐keto‐3‐methylvalerate were analyzed by UV detector at 215 nm.

### CulECpy Construction and Application

Co‐cultivated Enzyme Constraint metabolic network model (CulECpy) was a streamlined method developed for rapidly constructing enzyme‐constrained models of synthetic consortia, incorporating global flux balance analysis (eq. 1) and enzymatic kinetic constraints for modular strains (eq. 2).

(1) $$\begin{array}{c} S_{cul}\cdot v_{cul}=0\\ 0\leq v_{cul}\leq v_{u} \end{array}$$

(2) $$\sum_{i=1}^{n}\frac{v_{x\;i}\cdot MW_{x\;i}}{k_{cat,x\;i}}\leq ENZ_{x}$$

*x* is number/id of strain.

*S_cul_* represents the stoichiometric matrix of the new model, and *v_cul_* represents the fluxes vector of all reactions, since all reversible reactions were disassembled into irreversible reactions, the lower limit of the reaction fluxes was 0.

CulECpy was upgraded and revamped. NSGA‐II multi‐objective optimization algorithm, rooted in genetic algorithms, has been incorporated into the upgrading method. Novel linear programming constraints including synthetic consortium growth and key reactions have been introduced, which aimed to enhance the congruence between simulated outcomes and actual synthetic consortium. Original *E. coli* model (iML1515) was revised as the basis in CulECpy. The built‐in script of CulECpy can automatically detect external metabolites within the model, construct metabolic interactions, and achieve flux connection.

To enable rapid model reconstruction with different pathway allocation strategies, CulECpy was employed to evaluate these strategies, allowing pathway allocation strategies to be labeled according to various intermediate metabolites. Using 2MBA synthesis as an example, it identified the missing upstream and downstream reactions for 17 sets of intermediate metabolites. The analysis module uses this annotated information and the complete dual‐strain model as inputs for simulation analysis. A multi‐objective optimization approach was employed to ensure basic biomass synthesis for both strain modules using lower‐bound constraints. The model simulated flux distributions to maximize 2MBA production, using the resulting maximum 2MBA synthesis flux under each strategy as a key evaluation criterion. Finally, the revised CulECpy function can directly output the results of flux balance analysis under enzyme constraints for various allocation strategies, as well as differences in upstream and downstream biomass synthesis.

### Statistical Analysis

All statistical analysis was performed using GraphPad Prism V9.5.1. One‐way analysis of variance (ANOVA) with Dunnett's multiple comparison tests was used for the comparison of more than two groups. All experiments were performed with three replicates unless specified, and the error bars in the figure legends represent means ± s.d. A *p* value less than 0.05 was considered statistically significant.

## Conflict of Interest

The authors declare no conflict of interest.

## Author Contributions

Y.L. and B.X. contributed equally to this work. Y.L. performed the experiments, collected and analyzed the data, and wrote the manuscript with input from all authors. B.X. designed the methodology, implemented the code, collected and analyzed the data, and contributed to wrote the manuscript with input from all authors. S.W. conceived and designed the study, contributed to the writing and editing of the manuscript. H.S. conceptualized the study, conceived and designed the research, and edited the manuscript.

## Supporting information



Supporting Information

## Data Availability

The data that support the findings of this study are available from the corresponding author upon reasonable request.
