# Supplementary material for: Model‐Guided Rational Construction of Escherichia coli Synthetic Consortia for Enhanced 2‐Methylbutyric Acid Production
Source: Adv Sci (Weinh). 2025 May 5;12(22):2416272. doi: 10.1002/advs.202416272 (PMC12165103; doi:10.1002/advs.202416272)
Supplement: Supplementary file 1 — Supporting Information [file ADVS-12-2416272-s001.docx]

**Model-guided rational construction of *Escherichia coli* synthetic consortia for enhanced 2-methylbutyric acid production**

Yu Liu^#^, Boyuan Xue^#^, Shaojie Wang*, Haijia Su*

State Key Laboratory of Green Biomanufacturing, National Energy R&D Center for Biorefinery, Beijing Key Laboratory of Green Chemicals Biomanufacturing, Beijing Synthetic Bio-manufacturing Technology Innovation Center, Beijing 102209, People’s Republic of China

#These authors contribute equally to this work.

*Corresponding author: suhj@mail.buct.edu.cn, wangshaojie@buct.edu.cn


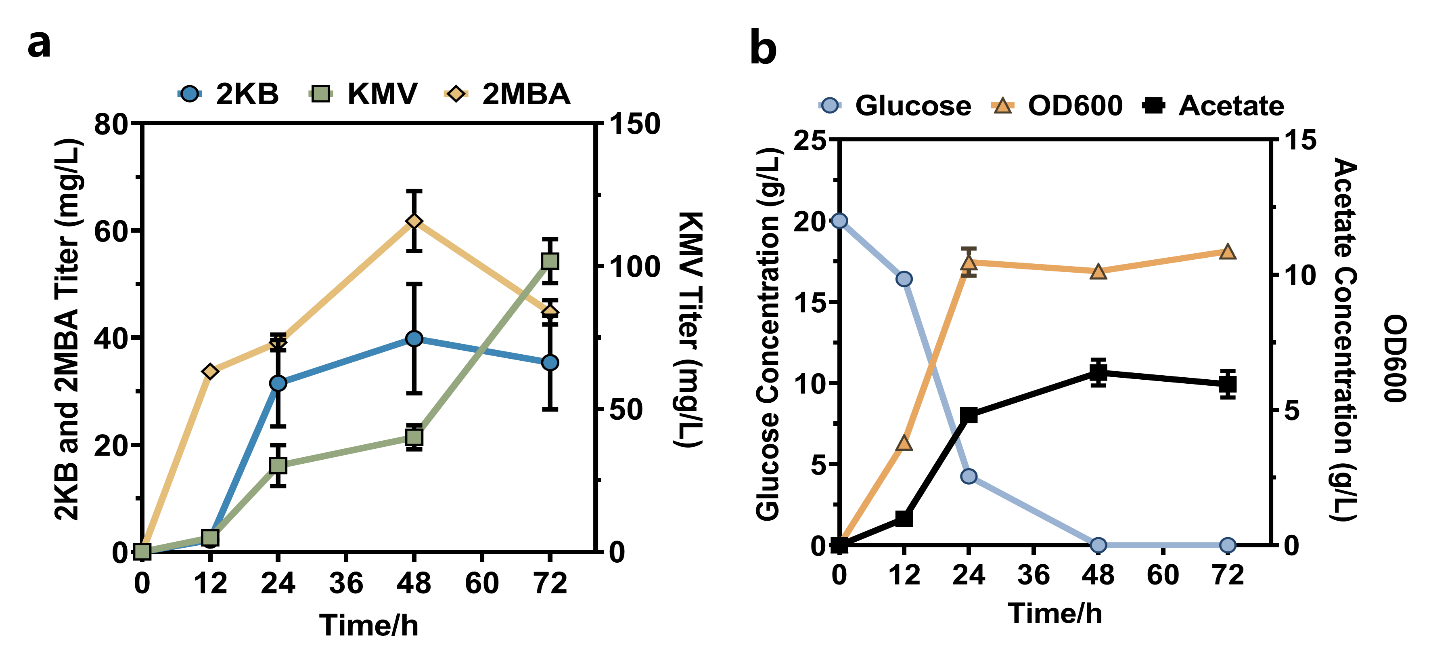


**Supplementary Figure 1** **2MBA biosynthesis via the citramalate pathway.** Temporal profiles of (**a**) 2KB, KMV, 2MBA and (**b**) cell growth, substrate degradation, acetate production during strain LY15 mono-culture. The mean ± s.d. of three biological replicates of a representative measurement is shown. All cells were grown and induced as described under experimental procedures.


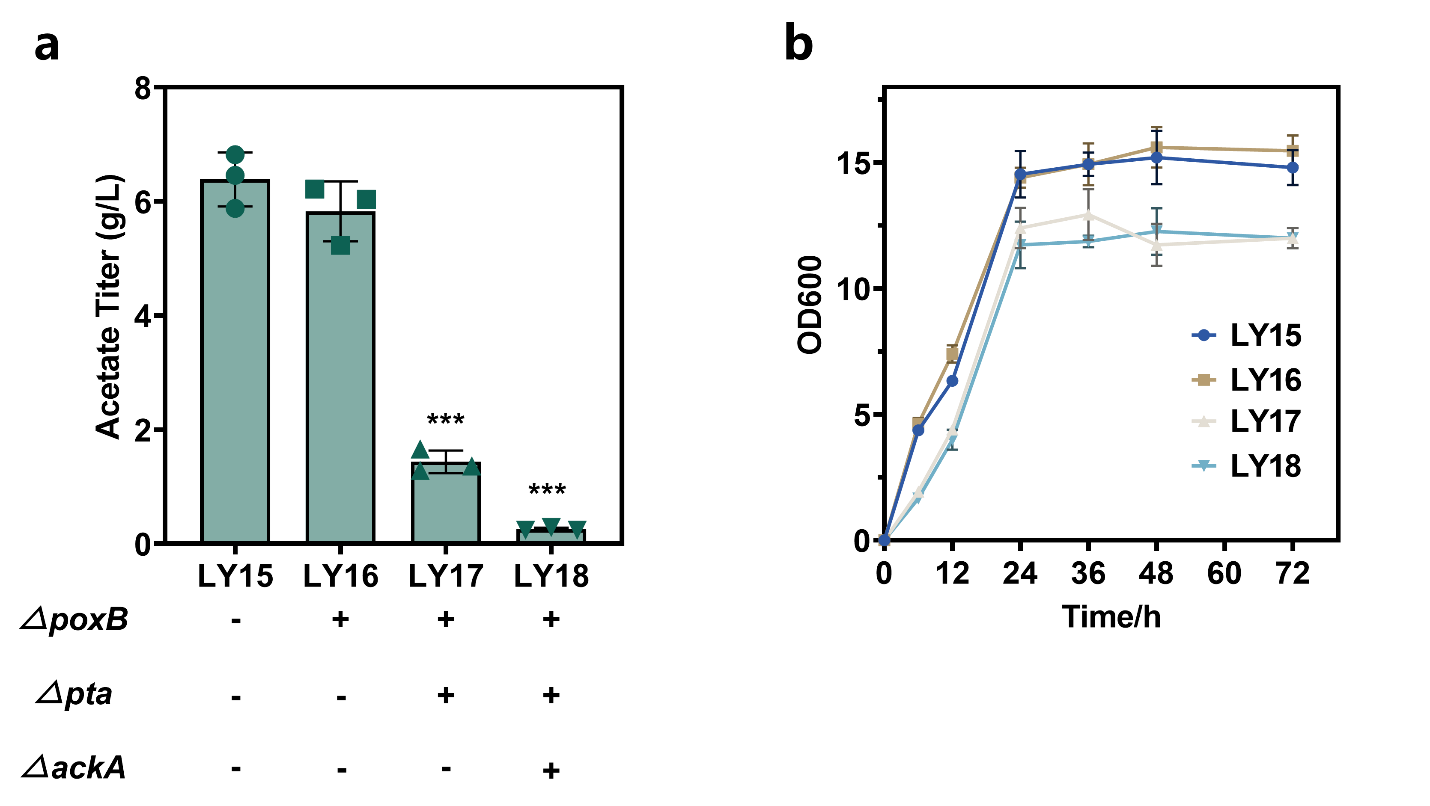


**Supplementary Figure 2 The impact of gradually deleting genes *poxB*, *pta*, and *ackA* on the strain LY16, LY17, and LY18.** (**a**) Acetate production in relevant strains. Statistical analysis was performed using a student’s t-test (one-tailed; * *p*＜0.05, ** *p*＜0.01 and *** *p*＜0.001; two-sample unequal variance). The mean ± s.d. of three biological replicates of a representative measurement is shown. All cells were grown and induced as described under experimental procedures. (**b**) Temporal profiles of cell growth during strain LY15, LY16, LY17, and LY18 mono-culture. The mean ± s.d. of three biological replicates of a representative measurement is shown. All cells were grown and induced as described under experimental procedures.


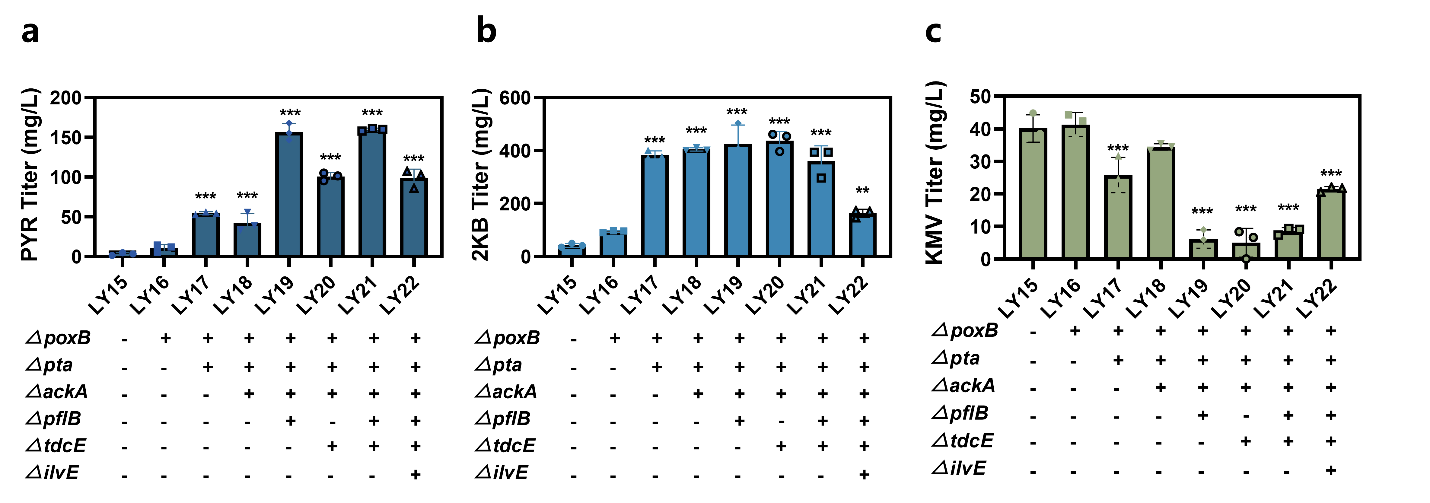


**Supplementary Figure 3 (a) PYR, (b) 2KB and (c) KMV production in relevant strains with competitive genes deletion.** Statistical analysis was performed using a student’s t-test (one-tailed; * *p*＜0.05, ** *p*＜0.01 and *** *p*＜0.001; two-sample unequal variance). The mean ± s.d. of three biological replicates of a representative measurement is shown. All cells were grown and induced as described under experimental procedures.


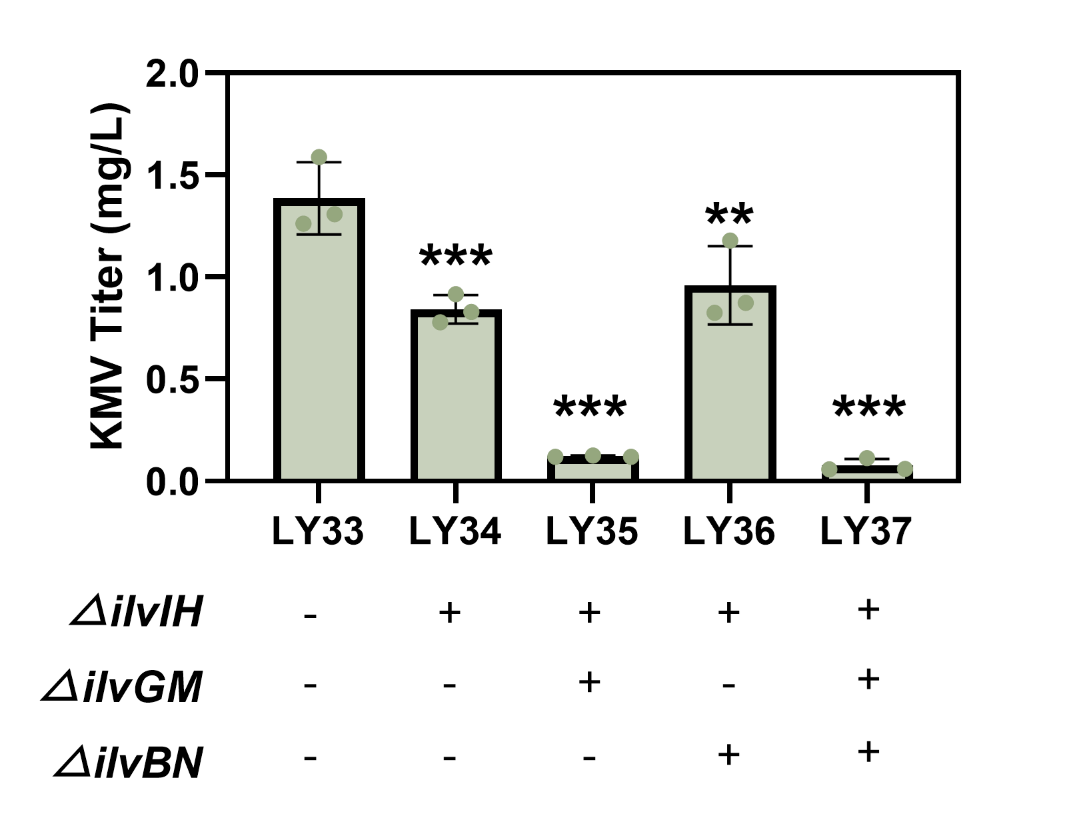


**Supplementary Figure 4** **KMV titer in the strains with different genes deletion encoding AHAS.** Statistical analysis was performed using a student’s t-test (one-tailed; * *p*＜0.05, ** *p*＜0.01 and *** *p*＜0.001; two-sample unequal variance). All cells were grown and induced as described under experimental procedures. The mean ± s.d. of three biological replicates of a representative measurement is shown.


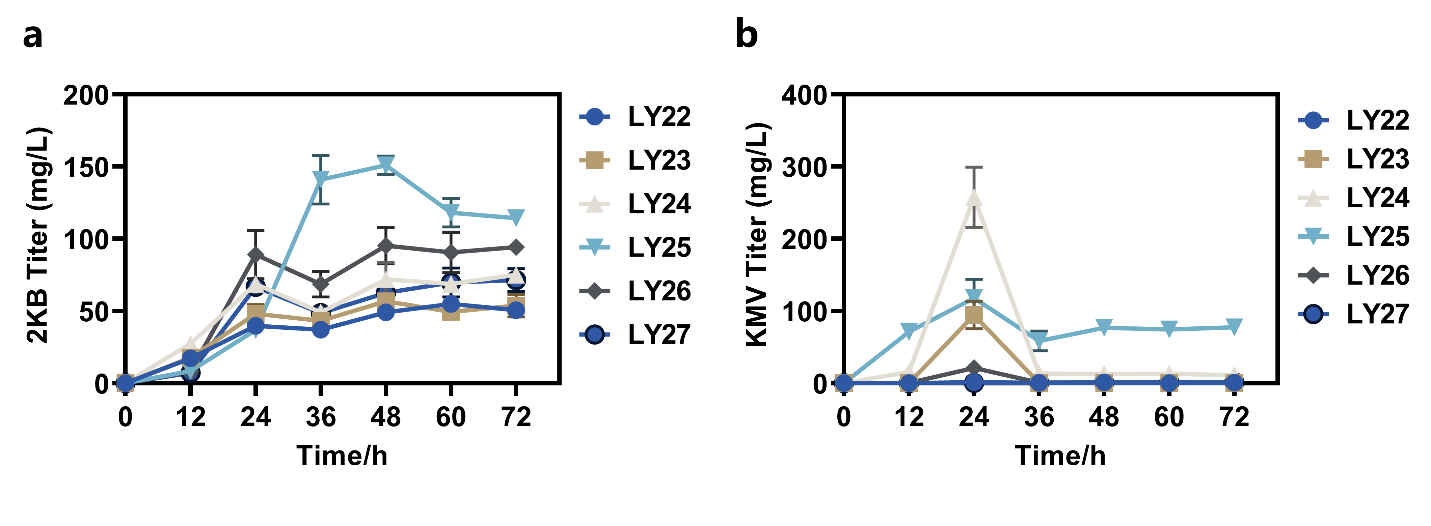


**Supplementary Figure 5 The overexpression of genes in branched-chain ketoacid synthesis module and its impact on 2KB and KMV synthesis.** Temporal profiles of (**a**) 2KB and (**b**) KMV production in the strain LY23, LY24, LY25, LY26, and LY27 with *ilvG*, *ilvM*, *ilvC*, and *ilvD* overexpression. All cells were grown and induced as described under experimental procedures. The data are presented as the average of three independent experiments, and error bars indicate standard errors.


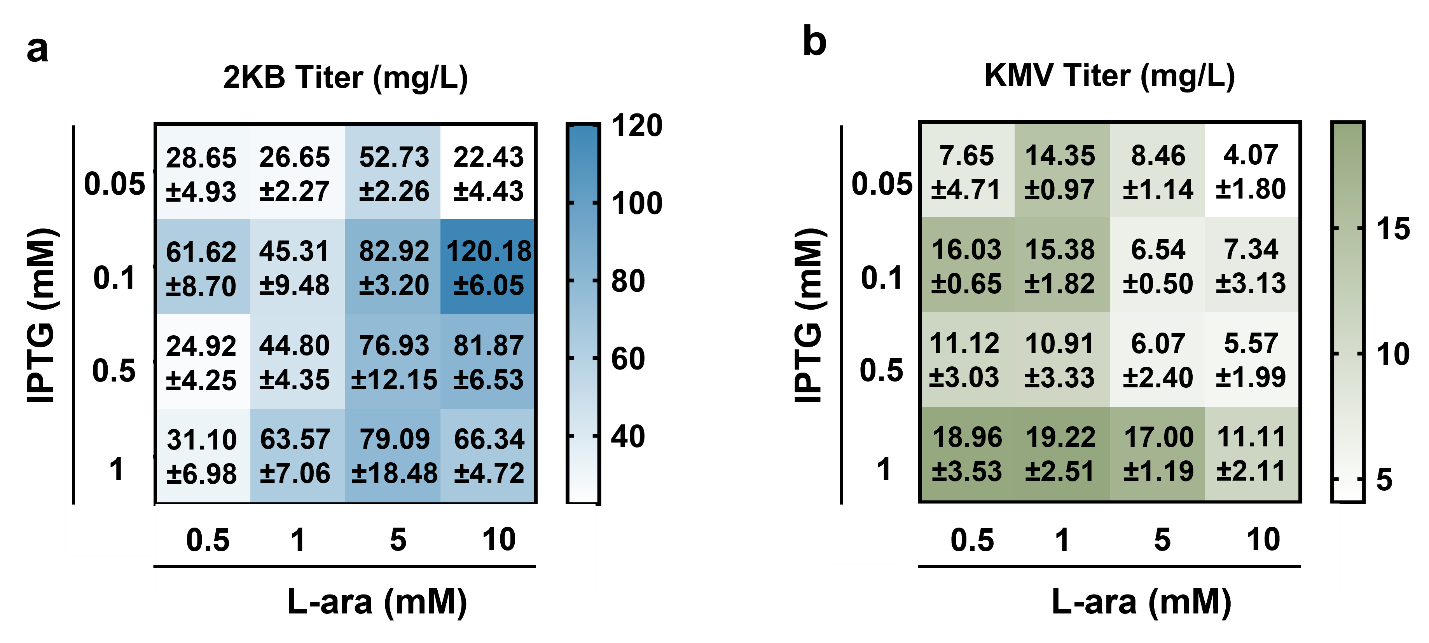


**Supplementary Figure 6 Titer of (a) 2KB and (b) KMV in orthogonal experiments testing various concentrations of L-arabinose and IPTG in 2MBA biosynthesis.** All cells were grown and induced as described under experimental procedures. The mean ± s.d. of three biological replicates of a representative measurement is shown.


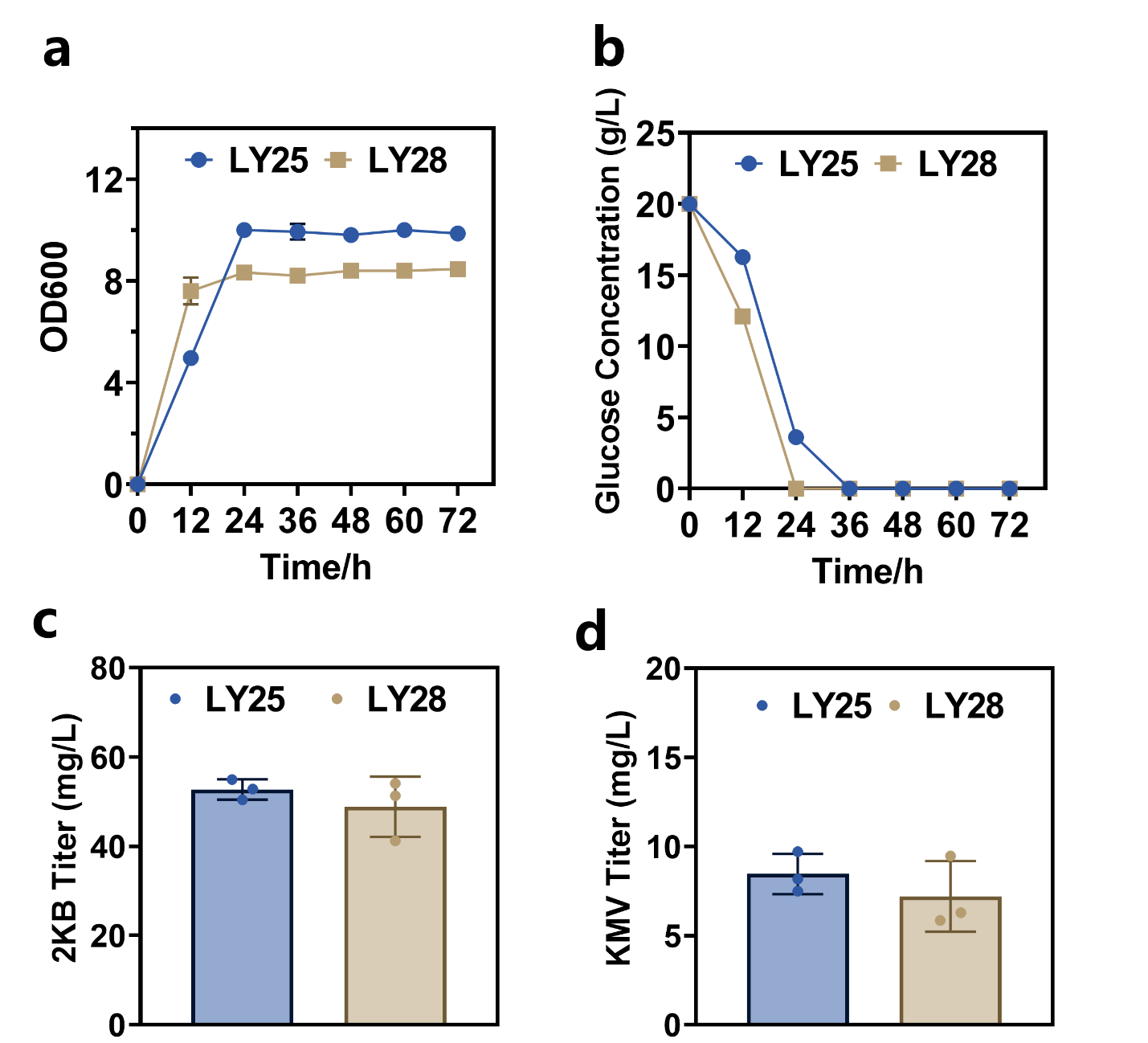


**Supplementary Figure 7 Comparison of (a) cell growth, (b) glucose consumption, (c) 2KB, and (d) KMV between single-plasmid strain LY25 and double-plasmid strain LY28 in the 2MBA biosynthesis.** Statistical analysis was performed using a student’s t-test (one-tailed; * *p*＜0.05, ** *p*＜0.01 and *** *p*＜0.001; two-sample unequal variance). All cells were grown and induced as described under experimental procedures. The mean ± s.d. of three biological replicates of a representative measurement is shown.


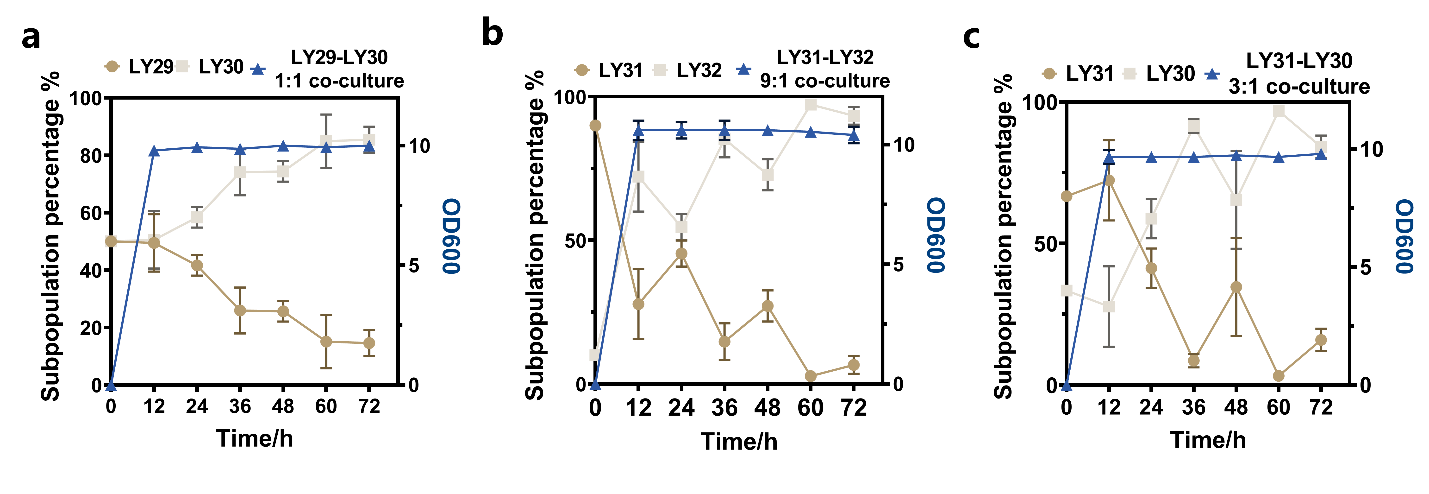
**Supplementary Figure 8 The dynamic growth and 2MBA biosynthesis behaviors of two-strain co-culture cultivated in shake flask.** Temporal profiles of the co-culture cell density change (blue lines) and individual subpopulation percentage change (brown and beige lines) of (**a**) LY29-LY30, (**b**) LY31-LY32, and (**c**) LY31-LY30. The data are presented as the average of three independent experiments, and error bars indicate standard errors.

**Supplementary Table 1 Primers used in this study**

| Primer | Sequence (5´-3´) |
| --- | --- |
| pE8a bankbone-F | CCAAAGGATCCAAACTCGAG |
| pE8a bankbone-R | ATGTATATCTCCTTCTTAAAAGATCTTTTGAATTC |
| cimA-F | TTTAAGAAGGAGATATACATATGATGGTGCGCATTTTTGA |
| cimA-R | TTAGAGTTTACCCGTCACTTCGC |
| leuBCD-F | AAGTGACGGGTAAACTCTAAAGGAGGAATAAACCATGTCGAAG |
| leuBCD-R | CTCGAGTTTGGATCCTTTGGATCCTTAATTCATAAACGCAGGTTG |
| pACYCduet bankbone-F | GGATCCGAATTCGAGCTC |
| pACYCduet bankbone-R | GGTATATCTCCTTATTAAAGTTAAACAAAATTATTTCT |
| kivD-F | ACTTTAATAAGGAGATATACCATGTATACCGTGGGCGAC |
| kivD-R | ATATCTCCTTTTAACTTTTGTTCTGCTCGGC |
| ALD-F | CAAAAGTTAAAAGGAGATATACCATGCCGACGCTGTATACC |
| ALD-R | CAGGCGCGCCGAGCTCGAATTCGGATCCTTAGTTATCCAGACTCAGATTGATGTG |
| ilvGM-F | CTTTAATAAGGAGATATACCATGAATGGCGCACAGTGG |
| ilvGM-R | CTGTGGTGATGATGGTGATGTCAGGCGCGGATTTGTTG |
| ilvC-F | GAATGATCAAGGAGATATACATATGGCTAACTACTTCAATACACTGA |
| ilvC-R | TATATCTCCTTGCGCATTGATTAACCCGCAACAGCAATACG |
| ilvD-F | TCAATGCGCAAGGAGATATACATATGCCTAAGTACCGTTCCG |
| ilvD-R | CTGTGGTGATGATGGTGATGTTAACCCCCCAGTTTCGATTTATC |
| pEcgRNA bankbone-F | TCTAGAGTCGACCTGCAG |
| pEcgRNA bankbone-F | ACTAGTATTATACCTAGGACTGAGCTAGC |
| poxB N20-F | GTCCTAGGTATAATACTAGTTGTCGGCGTTCATCATGGTAGTTTTAGAGCTAGAAATAGCAAGTT |
| poxB N20-R | TTCTGCAGGTCGACTCTAGAGAATTCAAAAAAAGCACCGACTC |
| poxB upstream-F | GGTTTTAACGCCACGTCG |
| poxB upstream-R | CCTTCTCCATATGAAACAAACGGTTGCAG |
| poxB downstream-F | GTTTGTTTCATATGGAGAAGGCGCGTTGC |
| poxB downstream-R | AAACCGAGCGAGAAAGCCATTCA |
| pEcgRNA bankbone-F | TCTAGAGTCGACCTGCAG |
| pEcgRNA bankbone-F | ACTAGTATTATACCTAGGACTGAGCTAGC |
| pta N20-F | GTCCTAGGTATAATACTAGTCGTTAACAAACTGAACGCACGTTTTAGAGCTAGAAATAGCAAGTT |
| pta N20-R | TTCTGCAGGTCGACTCTAGAGAATTCAAAAAAAGCACCGACTC |
| pta upstream-F | TCCCAACCAACGAAGAACT |
| pta upstream-R | CGTAAACGTAAAAGTTCAGAGACTGGGCAAA |
| pta downstream-F | GTCTCTGAACTTTTACGTTTACGGTGACTGTG |
| pta downstream-R | TTACTGCTGCTGTGCAGA |
| ackA N20-F | GTCCTAGGTATAATACTAGTTCTGCGCAGCTGACTGCTATGTTTTAGAGCTAGAAATAGCAAGTT |
| ackA N20-R | TTCTGCAGGTCGACTCTAGAGAATTCAAAAAAAGCACCGACTC |
| ackA upstream-F | GTCTTCCATACCCACTATCAGG |
| ackA upstream-R | AACGTCCATTGAGTTCTGGTTTTTGTGCCAG |
| ackA downstream-F | ACCAGAACTCAATGGACGTTTACTGCCAC |
| ackA downstream-R | GCTGAGCGATAGGTTTGAAAAC |
| pflB N20-F | GTCCTAGGTATAATACTAGTTTACGGCAACGCAGGATGTCGTTTTAGAGCTAGAAATAGCAAGTT |
| pflB N20-R | TTCTGCAGGTCGACTCTAGAGAATTCAAAAAAAGCACCGACTC |
| pflB upstream-F | ATGTCCGAGCTTAATGAAAAGTTAG |
| pflB upstream-R | GCGTCACGGTGTCAGAACACCAGATTTACGG |
| pflB downstream-F | GTGTTCTGACACCGTGACGCTATCCCGA |
| pflB downstream-R | TTACATAGATTGAGTGAAGGTACGAG |
| tdcE N20-F | GTCCTAGGTATAATACTAGTTATTTCGCCGCCATTTCCTGGTTTTAGAGCTAGAAATAGCAAGTT |
| tdcE N20-R | TTCTGCAGGTCGACTCTAGAGAATTCAAAAAAAGCACCGACTC |
| tdcE upstream-F | AATAATCGCTCTAACTCCTGTGG |
| tdcE upstream-R | GTATTACCGGGTTTGCAGGCCAACAATTT |
| tdcE downstream-F | GCCTGCAAACCCGGTAATACGCCGGACG |
| tdcE downstream-R | ACATAAGGGCCGATTGCG |
| ilvE N20-F | GTCCTAGGTATAATACTAGTATCCGTCCGCTGATCTTCGTGTTTTAGAGCTAGAAATAGCAAGTT |
| ilvE N20-R | TTCTGCAGGTCGACTCTAGAGAATTCAAAAAAAGCACCGACTC |
| ilvE upstream-F | CCACCCTTACCGATAACCC |
| ilvE upstream-R | TTTCGCCAGTGAAGTTTTATATTCCTTTTGCGCTCAGG |
| ilvE downstream-F | GGAATATAAAACTTCACTGGCGAAACCGA |
| ilvE downstream-R | AGGGGTGATTTTGTCGCA |
| ilvIH N20-F | GTCCTAGGTATAATACTAGTGCTTTCTGGCTGGCGGCAAGGTTTTAGAGCTAGAAATAGCAAGTT |
| ilvIH N20-R | TTCTGCAGGTCGACTCTAGAGAATTCAAAAAAAGCACCGACTC |
| ilvIH upstream-F | CAAACGCATTTGGTCGCT |
| ilvIH upstream-R | TGAGATCATTCCACTGTTTGACGGAAAAAATGTGTAAA |
| ilvIH downstream-F | GTCAAACAGTGGAATGATCTCAATGCGCAATTT |
| ilvIH downstream-R | CATTTCGTTGTCTGGCTGAT |
| ilvGM N20-F | GTCCTAGGTATAATACTAGTCAAAAACCGATGCTGTACGTGTTTTAGAGCTAGAAATAGCAAGTT |
| ilvGM N20-R | TTCTGCAGGTCGACTCTAGAGAATTCAAAAAAAGCACCGACTC |
| ilvGM upstream-F | AACTACGAGGAAGGGAACAAC |
| ilvGM upstream-R | GTCCACCAGTTGATAACCGAAAACGGTGTTC |
| ilvGM downstream-F | TTCGGTTATCAACTGGTGGACGTCGCAC |
| ilvGM downstream-R | TGCTCCAGCGCTTCTGCG |
| ilvBN N20-F | GTCCTAGGTATAATACTAGTCAGATAAAGCACCGGGCGTTGTTTTAGAGCTAGAAATAGCAAGTT |
| ilvBN N20-R | TTCTGCAGGTCGACTCTAGAGAATTCAAAAAAAGCACCGACTC |
| ilvBN upstream-F | TACCAGCCGCAGGCGACT |
| ilvBN upstream-R | GCTGCACTTTCAGGATAGAACCGCCCGG |
| ilvBN downstream-F | GTTCTATCCTGAAAGTGCAGCGTAATCAGT |
| ilvBN downstream-R | ATAACAGCCGCCCGTGGC |

**Supplementary Table 2 Plasmids used in this study.**

| Plasmids | Description | Sourse |
| --- | --- | --- |
| pE8a | araBAD promoter, Amp^R^ | Novagen |
| pACYCduet-1 | double T7 promoters, Cm^R^ | Novagen |
| p01 | pE8a carrying *cimA, leuB*, *leuC*, *leuD*, Amp^R^ | This study |
| p02 | pACYCduet carrying *kivD* and *ALD2*, Cm^R^ | This study |
| p03 | pACYCduet carrying *ilvC*, *kivD*, *ALD2*, Cm^R^ | This study |
| p04 | pACYCduet carrying *ilvD*, *kivD*, *ALD2*, Cm^R^ | This study |
| p05 | pACYCduet carrying *ilvG*, *ilvM*, *kivD*, *ALD2*, Cm^R^ | This study |
| p06 | pACYCduet carrying *ilvG*, *ilvM*, *ilvC*, *kivD*, *ALD2*, Cm^R^ | This study |
| p07 | pACYCduet carrying *ilvG*, *ilvM*, *ilvC*, *ilvD*, *kivD*, *ALD2*, Cm^R^ | This study |
| p08 | pE8a carrying *cimA*, *leuB*, *leuC*, *leuD*, *ilvG*, *ilvM*, *kivD*, *ALD2*, Amp^R^ | This study |
| p09 | pE8a carrying *cimA*, *leuB*, *leuC*, *leuD* and kanamycin resistance gene, Amp^R^ and Kan^R^ | This study |
| p10 | pE8a carrying *ilvG*, *ilvM*, *kivD*, *ALD2* and spectinomycin resistance gene, Amp^R^ and Spe^R^ | This study |
| P11 | pE8a carrying *cimA*, *leuB*, *leuC*, *leuD*, *kivD*, *ALD2* and kanamycin resistance gene, Amp^R^ and Kan^R^ | This study |
| P12 | pE8a carrying *kivD, ALD2*, and spectinomycin resistance gene, Amp^R^ and Spe^R^ | This study |
| pEcgRNA | Derived from pTargetF, ccdB | ^46^ |
| pEcCas | Derived from pCas, sacB, PrhaB-sgRNA-pMB1, pSC101 | ^46^ |
| pEcΔpoxB | Derived from pEcgRNA, target *poxB* in *E. coli* BL21(DE3) | This study |
| pEcΔpta | Derived from pEcgRNA, target *pta* in *E. coli* BL21(DE3) | This study |
| pEcΔackA | Derived from pEcgRNA, target *ackA* in *E. coli* BL21(DE3) | This study |
| pEcΔpflB | Derived from pEcgRNA, target *pflB* in *E. coli* BL21(DE3) | This study |
| pEcΔtdcE | Derived from pEcgRNA, target *tdcE* in *E. coli* BL21(DE3) | This study |
| pEcΔilvE | Derived from pEcgRNA, target *ilvE* in *E. coli* BL21(DE3) | This study |
| pEcΔilvIH | Derived from pEcgRNA, target *ilvIH* in *E. coli* BL21(DE3) | This study |
| pEcΔilvGM | Derived from pEcgRNA, target *ilvGM* in *E. coli* BL21(DE3) | This study |
| pEcΔilvBN | Derived from pEcgRNA, target *ilvBN* in *E. coli* BL21(DE3) | This study |

**Supplementary Table 3 *E. coli* strains used in this study.**

| Strains | Description | Sourse |
| --- | --- | --- |
| LY01 | *E. coli* BL21(DE3) F^-^ *omp*T *hsd*S_B_ (r_B_^–^, m_B_^–^) *gal* *dcm* (DE3) | TransGen Biotech |
| LY02 | LY01 *△poxB* | This study |
| LY03 | LY01 *△poxB△pta* | This study |
| LY04 | LY01 *△poxB△pta△*ackA | This study |
| LY05 | LY01 *△poxB△pta△*ackA*△pflB* | This study |
| LY06 | LY01 *△poxB△pta△*ackA*△tdcE* | This study |
| LY07 | LY01 *△poxB△pta△*ackA*△pflB△tdcE* | This study |
| LY08 | LY01 *△poxB△pta△*ackA*△pflB△tdcE△ilvE* | This study |
| LY09 | LY01 *△poxB△pta△*ackA*△ilvIH* | This study |
| LY10 | LY01 *△poxB△pta△*ackA*△ilvIH△ilvGM* | This study |
| LY11 | LY01 *△poxB△pta△*ackA*△ilvIH△ilvBN* | This study |
| LY12 | LY01 *△poxB△pta△*ackA*△ilvIH△ilvGM△ilvBN* | This study |
| LY15 | LY01 carrying p01 and p02 | This study |
| LY16 | LY02 carrying p01 and p02 | This study |
| LY17 | LY03 carrying p01 and p02 | This study |
| LY18 | LY04 carrying p01 and p02 | This study |
| LY19 | LY05 carrying p01 and p02 | This study |
| LY20 | LY06 carrying p01 and p02 | This study |
| LY21 | LY07 carrying p01 and p02 | This study |
| LY22 | LY08 carrying p01 and p02 | This study |
| LY23 | LY08 carrying p01 and p03 | This study |
| LY24 | LY08 carrying p01 and p04 | This study |
| LY25 | LY08 carrying p01 and p05 | This study |
| LY26 | LY08 carrying p01 and p06 | This study |
| LY27 | LY08 carrying p01 and p07 | This study |
| LY28 | LY08 carrying p08 | This study |
| LY29 | LY12 carrying p09 | This study |
| LY30 | LY08 carrying p10 | This study |
| LY31 | LY08 carrying p11 | This study |
| LY32 | LY08 carrying p12 | This study |
| LY33 | LY04 carrying p01 | This study |
| LY34 | LY09 carrying p01 | This study |
| LY35 | LY10 carrying p01 | This study |
| LY36 | LY11 carrying p01 | This study |
| LY37 | LY12 carrying p01 | This study |

**Supplementary Table 4 Codon optimized genes used in this study.**

| Synthesized genes | Sequence (5’-3’) |
| --- | --- |
| *cimA* | ATGATGGTGCGCATTTTTGACACCACGCTGCGCGATGGCGAACAGACCCCGGGCGTTAGTCTGACCCCGAACGACAAGCTGGAGATCGCCAAAAAGCTGGACGAGCTGGGCGTGGATGTTATCGAAGCCGGCAGCGCGGTTACCAGCAAGGGTGAACGCGAGGGCATCAAACTCATCACCAAAGAGGGTCTGAACGCCGAAATCTGCAGCTTCGTTCGCGCGCTGCCAGTTGACATTGATGCGGCGCTGGAATGCGATGTTGACAGCGTTCATCTGGTGGTTCCGACCAGTCCGATCCACATGAAATACAAGCTCCGCAAGACGGAAGATGAGGTTCTCGTGACGGCGCTGAAAGCGGTTGAGTACGCCAAAGAGCAAGGTCTGATCGTTGAGCTCAGCGCCGAAGACGCCACGCGTAGCGACGTGAACTTTCTGATCAAGCTCTTCAACGAGGGCGAGAAAGTTGGTGCCGACCGCGTGTGCGTGTGCGATACCGTTGGCGTTCTGACGCCGCAGAAGAGCCAAGAACTGTTCAAGAAAATCACGGAGAACGTGAACCTCCCGGTTAGCGTTCATTGCCACAACGACTTCGGCATGGCCACCGCCAATGCGTGCAGTGCGGTTCTGGGTGGTGCGGTGCAGTGCCATGTTACCGTTAACGGCATCGGCGAACGTGCCGGCAACGCGAGTCTGGAAGAAGTTGTTGCCGCGAGCAAGATTCTGTACGGCTACGACACGAAAATCAAGATGGAAAAGCTGTACGAGGTGAGTCGCATCGTTAGTCGCCTCATGAAACTGCCGGTGCCGCCGAATAAGGCCATCGTTGGCGACAACGCCTTCGCCCACGAAGCGGGTATCCATGTTGACGGTCTGATCAAGAACACCGAAACCTACGAGCCGATCAAACCGGAGATGGTTGGCAATCGTCGCCGCATCATTCTGGGCAAGCACAGCGGTCGCAAGGCGCTGAAGTATAAGCTCGATCTGATGGGCATCAACGTGAGCGACGAACAGCTGAACAAGATCTACGAGCGCGTTAAGGAGTTCGGCGATCTGGGCAAATACATCAGCGATGCCGATCTGCTGGCGATTGTGCGCGAAGTGACGGGTAAACTCTAA |
| *kivD* | ATGTATACCGTGGGCGACTATCTGCTCGATCGTCTGCACGAGCTGGGTATCGAAGAGATCTTTGGCGTGCCGGGCGACTACAACCTCCAGTTCCTCGACCAGATCATCAGCCGCAAGGACATGAAATGGGTGGGCAACGCGAATGAACTGAACGCCAGTTACATGGCGGACGGTTACGCGCGCACGAAGAAAGCCGCGGCCTTTCTCACCACCTTCGGCGTTGGCGAACTGAGTGCGGTTAATGGCCTCGCCGGTAGCTACGCGGAGAATCTGCCGGTTGTGGAAATCGTTGGCAGCCCAACCAGTAAGGTGCAGAACGAGGGTAAGTTCGTTCATCACACGCTGGCGGACGGCGATTTCAAGCACTTCATGAAGATGCATGAGCCGGTTACCGCCGCGCGTACGCTGCTCACCGCCGAAAACGCCACCGTGGAGATCGACCGCGTGCTGAGTGCGCTGCTGAAAGAGCGCAAGCCAGTTTACATCGATCTGCCAGTGGATGTTGCCGCGGCGAAAGCGGAAAAACCACTGCTGCCGCTGAAGAAGGAGAACCCGACGAGTAACACGAGCGACCAAGAAATTCTGAATAAAATCCAAGAAAGTCTCAAGAATGCGAAGAAGCCGATCGTGATTACGGGCCACGAGATCATTAGCTTCGGTCTGGAGAACACCGTGACCCAGTTCATCAGCAAGACCAAACTCCCGATCACGACGCTGAACTTCGGTAAGAGTAGCGTGGACGAAGCGCTGCCGAGCTTTCTGGGTATCTATAACGGCAAGCTGAGCGAGCCGAATCTGAAGGAGTTTGTGGAGAGTGCGGACTTCATCCTCATGCTGGGTGTTAAGCTCACCGATAGCAGCACGGGTGCCTTCACGCACCATCTGAACGAGAATAAGATGATTAGTCTGAACATCGACGAGGGCAAGATCTTCAACGAGCGCATCCAAAATTTCGACTTCGAGAGCCTCATTAGCAGCCTCCTCGATCTGAGCGAGATCGAGTACAAGGGCGACTACATCGACAAGAAGCAAGAAAATTTCGTTCCGAGCAACGCGCTGCTCAGTCAAGATCGTCTCTGGCAAGCCGTTGAGAGTCTGACCCAAAGCAACGAAACGATCGTTGCCGAACAAGGCACCAGCTTCTTCGGCGCGAGCAGCATCTTTCTGAAGCCGAAGAGCCACTTTATCGGCCAGCCACTGTGGGGTAGCATCGGTTATACCTTTCCGGCGGCGCTGGGCAGTCAGATTGCCGATAAGGAGAGCCGCCATCTGCTGTTCATCGGTGACGGTAGTCTGCAGCTCACCGTTCAAGAACTGGGTCTGGCCATCCGCGAAAAAATCAACCCGATCTGCTTTATCATCAACAACGACGGCTACACGGTGGAGCGTGAGATCCACGGTCCGAATCAGAGCTACAACGACATCCCGATGTGGAACTACAGTAAGCTGCCGGAGAGTTTTGGCGCGACCGAGGATCGTGTGGTGAGTAAGATCGTGCGCACCGAGAACGAGTTCGTTAGCGTGATGAAGGAGGCGCAAGCCGATCCGAATCGCATGTACTGGATCGAGCTCATTCTGGCGAAAGAGGACGCCCCGAAGGTGCTGAAGAAGATGGGCAAGCTGTTCGCCGAGCAGAACAAAAGTTAA |
| *ALD2* | ATGCCGACGCTGTATACCGACATCGAAATCCCGCAGCTGAAGATTAGTCTGAAGCAGCCGCTGGGTCTGTTCATCAACAATGAGTTCTGCCCGAGCAGCGACGGCAAAACCATTGAAACCGTTAACCCGGCCACGGGCGAACCGATCACCAGCTTCCAAGCCGCGAATGAGAAGGACGTGGACAAAGCGGTTAAGGCGGCGCGTGCCGCCTTCGACAATGTGTGGAGCAAGACCAGTAGCGAACAGCGCGGCATCTATCTGAGCAATCTGCTGAAGCTGATCGAGGAGGAACAAGATACGCTGGCGGCGCTGGAGACGCTGGATGCGGGCAAACCATACCACAGCAACGCGAAGGGCGATCTGGCCCAAATTCTGCAGCTCACCCGCTATTTCGCGGGTAGCGCGGACAAGTTTGACAAAGGCGCCACCATCCCGCTGACCTTCAATAAGTTCGCCTACACGCTGAAGGTGCCATTCGGCGTGGTTGCCCAGATCGTTCCGTGGAACTATCCACTGGCGATGGCGTGTTGGAAACTGCAAGGCGCGCTGGCCGCCGGTAATACCGTGATTATTAAGCCAGCCGAGAACACCAGTCTCAGTCTGCTGTACTTTGCGACGCTGATCAAGAAAGCGGGTTTCCCGCCGGGCGTGGTGAATATCGTTCCGGGCTACGGTAGCCTCGTTGGCCAAGCGCTCGCGAGTCACATGGACATCGACAAGATTAGCTTCACCGGCAGCACGAAGGTTGGTGGCTTTGTTCTGGAAGCGAGTGGCCAGAGCAATCTGAAGGACGTGACGCTGGAATGCGGTGGCAAAAGCCCAGCGCTGGTTTTTGAAGACGCCGATCTGGACAAGGCCATTGATTGGATCGCCGCCGGTATCTTCTACAACAGCGGCCAGAATTGCACGGCGAATAGTCGCGTGTACGTGCAGAGCAGCATCTACGACAAATTCGTGGAAAAGTTCAAGGAAACCGCGAAGAAGGAGTGGGATGTGGCCGGCAAGTTCGACCCATTCGACGAGAAGTGCATTGTGGGCCCGGTGATCAGCAGCACGCAATACGATCGCATCAAGAGTTATATTGAGCGCGGCAAACGCGAAGAGAAGCTGGACATGTTCCAGACGAGCGAGTTTCCGATCGGCGGCGCCAAAGGCTACTTCATCCCGCCGACCATCTTCACCGATGTTCCGCAGACCAGCAAGCTGCTGCAAGATGAAATCTTTGGCCCGGTTGTGGTGGTGAGCAAGTTCACCAACTACGACGATGCGCTGAAGCTGGCGAACGACACGTGCTATGGTCTGGCGAGCGCGGTGTTCACCAAAGACGTGAAGAAGGCGCACATGTTCGCGCGCGACATCAAAGCGGGTACCGTGTGGATCAACAGCAGCAACGACGAGGACGTGACCGTGCCATTCGGTGGCTTCAAAATGAGCGGCATTGGTCGCGAGCTGGGTCAGAGCGGTGTGGATACCTATCTGCAAACCAAGGCGGTTCACATCAATCTGAGTCTGGATAACTAA |

**Supplementary Table 5 Reactions Associated with Model Pathway Allocation**

| No. | IDs | Equations |
| --- | --- | --- |
| 1 | PGI | D-Glucose 6-phosphate <=> D-Fructose 6-phosphate |
| 2 | PFK | ATP + D-Fructose 6-phosphate --> ADP + D-Fructose 1,6-bisphosphate + H^+^ |
| 3 | FBA | D-Fructose 1,6-bisphosphate <=> Dihydroxyacetone phosphate + Glyceraldehyde 3-phosphate |
| 4 | GAPD | Glyceraldehyde 3-phosphate + Nicotinamide adenine dinucleotide + Phosphate <=> 3-Phospho-D-glyceroyl phosphate + H^+^ + Nicotinamide adenine dinucleotide - reduced |
| 5 | PGK | 3-Phospho-D-glycerate + ATP <=> 3-Phospho-D-glyceroyl phosphate + ADP |
| 6 | PGM | D-Glycerate 2-phosphate <=> 3-Phospho-D-glycerate |
| 7 | ENO | D-Glycerate 2-phosphate <=> H_2_O + Phosphoenolpyruvate |
| 8 | PYK | ADP + H^+^ + Phosphoenolpyruvate --> ATP + Pyruvate |
| 9 | CIMA | Acetyl-CoA + H_2_O +Pyruvate --> Citramalate + Coenzyme A + H^+^ |
| 10 | LEUC | Citramalate <=> Citraconate + H_2_O |
| 11 | LEUD | Citraconate + H_2_O --> D-erythro-3-Methylmalate |
| 12 | LEUB | Nicotinamide adenine dinucleotide + D-erythro-3-Methylmalate --> 2-Oxobutanoate + CO_2_ + Nicotinamide adenine dinucleotide - reduced |
| 13 | ACHBS | 2-Oxobutanoate + H^+^ + Pyruvate --> (S)-2-Aceto-2-hydroxybutanoate + CO_2_ |
| 14 | KARA2 | (S)-2-Aceto-2-hydroxybutanoate + H^+^ + Nicotinamide adenine dinucleotide phosphate - reduced <=> (R)-2,3-Dihydroxy-3-methylpentanoate + Nicotinamide adenine dinucleotide phosphate |
| 15 | DHAD2 | (R)-2,3-Dihydroxy-3-methylpentanoate --> (S)-3-Methyl-2-oxopentanoate + H_2_O |
| 16 | KIVD | (S)-3-Methyl-2-oxopentanoate + H^+^ --> 2-methylbutanal + CO_2_ |
| 17 | ALD2 | 2-methylbutanal + H_2_O + Nicotinamide adenine dinucleotide --> 2-methylbutyric acid + H^+^ + Nicotinamide adenine dinucleotide - reduced |
